# Development of Skimmed Goat Milk Functional Ingredient Enriched with Grape Pomace Seed and *Agrocybe aegerita* Extracts: Optimization, Characterization and Application in Dehydrated Foods

**DOI:** 10.3390/foods15132397

**Published:** 2026-07-06

**Authors:** Ana Plećić, Danijel D. Milinčić, Ivana Sredović Ignjatović, Jovana Petrović, Aleksandar Ž. Kostić, Ana Doroški Petković, Steva M. Lević, Slađana P. Stanojević, Vladimir B. Pavlović, Vladislav Rac, Viktor A. Nedović, Mirjana B. Pešić

**Affiliations:** 1 Department of Food Technology and Biochemistry, Faculty of Agriculture, University of Belgrade, Nemanjina 6, 11080 Belgrade, Serbia; ana.bjekovic@agrif.bg.ac.rs (A.P.); danijel.milincic@agrif.bg.ac.rs (D.D.M.); isredovic@agrif.bg.ac.rs (I.S.I.); akostic@agrif.bg.ac.rs (A.Ž.K.); ana.doroski@agrif.bg.ac.rs (A.D.P.); slevic@agrif.bg.ac.rs (S.M.L.); sladjas@agrif.bg.ac.rs (S.P.S.); vlaver@agrif.bg.ac.rs (V.B.P.); vladarac@agrif.bg.ac.rs (V.R.); 2 Department of Plant Physiology, National Institute of Republic of Serbia, Institute for Biological Research “Siniša Stanković”, University of Belgrade, 11060 Belgrade, Serbia; jovana0303@ibiss.bg.ac.rs

**Keywords:** goat milk, grape pomace seed extract, mushroom extract, central composite design, antioxidant activity, functional ingredient, dehydrated soup

## Abstract

The aim of this study was to formulate and optimize a novel functional ingredient based on thermally treated skimmed goat milk enriched with *Agrocybe aegerita* mushroom extract (ME) and grape pomace seed extract (GPE), intended for application in a dehydrated soup model. A central composite design was applied for preliminary optimization and the formulation based on antioxidant properties. The optimized ingredient exhibited enhanced antioxidant activity, with GPE identified as the dominant factor influencing the responses. However, deviations between predicted and experimental values were observed, reflecting moderate model fitting and differences in assay mechanisms. ATR-FTIR spectra were dominated by milk compounds, while DLS and electrophoretic analysis revealed structural modifications, including polymodal particle size distribution and alterations in the protein profile, indicating interactions between milk proteins, polyphenols, and mushroom-derived compounds. UHPLC-QToF-MS analysis confirmed a high content of grape-derived phenolic compounds. Following simulated gastrointestinal digestion, several phenolic compounds were detected in the soluble fraction, with catechin and ethyl gallate exhibiting the highest bioaccessibility (12.58% and 4.54%). The enriched ingredient showed modified techno-functional properties, including reduced emulsifying capacity but improved foaming behavior, which was attributed to protein structural changes and intermolecular interactions. Application in a dehydrated soup model demonstrated good solubility, stability, and high sensory acceptability without negative effects on flavor. Furthermore, the enriched soup showed enhanced antioxidant properties after simulated gastrointestinal digestion. The developed formulation represents a promising natural functional ingredient, combining enhanced bioactive properties with satisfactory technological performance.

## 1. Introduction

Modern consumers increasingly expect food to provide additional health benefits beyond basic nutritional value. Functional products, i.e., foods enriched with bioactive components (vitamins, minerals, antioxidants, probiotics, omega-3-fatty acids, phenolic compounds, etc.), have become the standard in the healthy nutrition market. As a result, a wide range of different food ingredients with broad bioactive effects are now being combined in product formulations. The bioactive compounds present in mushrooms and grape pomace (including grape seeds) are well known and have been extensively researched. Nevertheless, these compounds remain underutilized in the development of functional foods. This gap suggests an opportunity to incorporate mushroom and grape seed bioactives into novel functional food formulations. *Agrocybe aegerita* is a high-quality edible mushroom valued for its pleasant taste, unique aroma, and rich nutritional composition [1]. It has become the subject of a growing number of studies investigating its bioactive compounds and potential applications in functional foods. In our previous studies, we demonstrated that aqueous extracts of *A. aegerita* are rich in bioactive compounds, primarily phenolic acids (such as *p*-hydroxybenzoic, protocatechuic, gallic, 4-methoxybenzoic, and coumaric acids), organic acids (such as malic, citric, fumaric, azelaic, and pinellic acids), peptides and some fatty acids [2]. This mushroom also exhibits a broad spectrum of biological activities, the most notable being antimicrobial, antioxidant, anticancer, antitumor, anti-inflammatory, immunomodulatory and cytotoxic effects [2,3,4,5]. Moreover, Bains et al. [6] evaluated the antibiofilm activity of *A. aegerita*, which can be considered part of its immunomodulatory action (i.e., preventing the formation of pathogenic biofilms). Their results suggest that *A. aegerita* exerts an inhibitory effect on the formation of bacterial biofilms.

On the other hand, several scientific studies have focused on identifying and structural characterization of individual phenolic compounds in the grape pomace of the red grape variety Prokupac [7,8,9]. Grape pomace, and therefore its constituent grape seeds, is a proven valuable source of bioactive compounds, primarily phenolic compounds that positively influence human health and have potential applications mainly in the food and pharmaceutical industries [9,10,11,12,13]. The amount of phenolic compounds extractable from grapes varies across different parts of the fruit: the seeds contain approximately 60–70% of the total soluble phenolics, the skins around 28–35%, while the pulp contains only about 10% [14,15]. When it comes to grape seeds and their phenolic profile, the predominant compounds are phenolic acids (derivatives of hydroxybenzoic and hydroxycinnamic acids), flavan-3-ols (proanthocyanidins) and flavonols [7,16,17,18]. All of these phenolic compounds, isolated from grape seeds in numerous scientific studies, exhibit a variety of bioactive properties that contribute to positive health effects. These include antioxidant, antimicrobial, anticancer, anti-inflammatory and cardioprotective properties [11,14,19]. However, the incorporation of bioactive compounds from grape seeds and mushrooms into the food systems is challenging due to their susceptibility to food processing conditions such as pH, temperature, light and oxygen, as well as their low solubility, interactions with food constituents and sensory characteristics such as smell, taste and color [20]. Milk-based carrier systems can help overcome these limitations. Among milk-based matrices, goat milk proteins have already been used in several studies to produce a variety of functional products, additives and beverages [21,22,23,24,25,26,27]. In particular, these proteins have been combined with grape seeds, demonstrating that goat milk proteins can serve as effective carriers for polyphenols. Such goat milk–polyphenol formulations have demonstrated potential polyphenol bioaccessibility during the colonic phase of digestion [18]. Likewise, only recently was the first functional ingredient based on goat milk and mushroom extract introduced [5].

To date, a complex system incorporating goat milk, mushroom and grape pomace seed extracts has not been studied. Despite extensive research on the bioactive properties of *A. aegerita* extracts and phenolic compounds derived from grape pomace, their incorporation into powdered or dehydrated food matrices remains largely unexplored. Furthermore, there is a notable absence of data regarding their application in dehydrated food products, such as instant soups, where functional ingredients can provide added nutritional and health benefits. Therefore, the aim of this study was to develop and optimize a novel goat milk-based functional ingredient enriched with mushroom and grape pomace seed extracts using response surface methodology, and to comprehensively evaluate its structural, physicochemical, techno-functional properties and sensory acceptability through an integrated multi-technique approach. This approach aims to bridge the existing knowledge gap and support the development of a novel functional ingredient specifically designed for incorporation into dehydrated food systems. Through the use of a central composite design (CCD), the formulation was optimized based on antioxidant properties, followed by integration into an instant soup model and comprehensive sensory evaluation.

## 2. Materials and Methods

### 2.1. Materials

The fruiting bodies of the mushroom *A. aegerita* were obtained from the fungal collection of the Institute for Biological Research “Siniša Stanković”, National Institute of the Republic of Serbia, University of Belgrade, Serbia. The freeze-dried fruiting bodies were finely ground into a powder, which was used as the starting material for preparing an aqueous extract.

Grapes of the Prokupac variety, an indigenous Serbian grape cultivar with a long tradition of cultivation in the Župa wine region, were collected in the winery (“Wine house Milinčić”). Grape seeds were manually separated from the fermented pomace and ground in a coffee grinder (Bosch MKM 6003 UC, BSH Hausgeräte GmbH, Munich, Germany).

Fresh goat’s milk was obtained from a local dairy and immediately skimmed prior to use.

### 2.2. Preparation of Goat Milk, Mushroom and Grape Pomace Seed Extracts

The preparation of the aqueous extract of *A. aegerita* (ME) was described in a previous study by Petrović et al. [28]. Briefly, 10 g of mushroom powder was extracted with 200 mL of MilliQ water in an ultrasonic bath for 90 min at room temperature. The extraction was repeated three times under identical conditions. The combined aqueous extracts were filtered, freeze-dried, and stored at −20 °C until further use. Grape pomace seed extracts (GPE) were prepared as previously described by Pešić et al. [29]. Briefly, lyophilized grape pomace seeds (approximately 1 g) were extracted with 20 mL of acidified 80% aqueous methanol (0.1% HCl) for 1 h under continuous shaking at room temperature. The extraction procedure was repeated three times, and the obtained filtrates were combined. The solvent was removed under reduced pressure at 40 °C, and the dried extract was reconstituted in MilliQ water, then lyophilized and stored in a freezer at −20 °C until further use. Samples of skimmed goat milk were heat-treated (90 °C, 10 min) (TM) and prepared according to the methodology previously described by Pesic et al. [30]. Milk samples were spray-dried and stored in a freezer until further use. TM without ME and GPE was used as the control sample.

### 2.3. Design of Experiments—DoE Based on Central Composite Design—CCD

To test the influence of mushroom extract and grape pomace seed extract content on the functional properties of the functional ingredient, two factors (content of grape pomace seed and mushroom extracts) were investigated using response surface methodology (RSM) and central composite design (CCD).

As described by Milinčić et al. [5], the experimental design and methodology were implemented using a central composite design in MINITAB software ver. 16 (Minitab Inc., State College, PA, USA). The factors investigated were tested at three levels (low, medium and high). Levels of factors in CCD for product based on goat’s milk proteins with mushroom extract and grape pomace seed extract are shown in Table S1.

The full factorial design, comprising a total of 13 experimental runs with all dependent variables (TPC, ABTS*+, DPPH*, FRP and CHE) measured in triplicate, is presented in Table S2.

#### 2.3.1. Preparation of Milk/ME/GPE Optimal Mixtures

Spray-dried, thermally treated goat milk powder was reconstituted with MilliQ water (1:10 *w*/*v*, 10%) and the pH was adjusted to 6.50. After addition of ME and GPE, the pH was monitored using a calibrated pH meter and adjusted to 6.50 using 1 M HCl. After that, according to the factorial design, suggested amounts of lyophilized mushroom extract and lyophilized grape pomace seed extract were added to prepared milk. Mixtures were mixed for 1 h on a mechanical shaker (MX-RD-E, DLAB Scientific Co., Ltd., Beijing, China) and further used for analysis.

#### 2.3.2. Total Phenolic Content and Antioxidant Properties of Prepared Mixtures

The determination of total phenolic content and antioxidant assays (such as ABTS*+, DPPH*, FRP and CHE) of the prepared mixtures were described in our previous study [5].

Based on the results obtained from the proposed experimental design, the optimal composition of the milk/ME/GPE functional ingredient was determined and subsequently used to prepare the dehydrated soup.

### 2.4. Physicochemical Characterization of Functional Ingredient

#### 2.4.1. Glucan Content

Glucan content in milk/ME/GPE powder was evaluated using Megazyme β-Glucan Assay Kit (Megazyme International Ireland Ltd., Bray, Co. Wicklow, Irelan) (Yeast and Mushroom), which is suitable for the indirect measurement of 1.3:1.6-β-glucan in yeast and mushroom preparations (Product code: K-YBGL). The procedure for total glucan and α-glucan content determination was performed according to manufacturer guidelines. The obtained values were calculated using the Mega-Calc software tool (Megazyme International Ireland Ltd., Bray, Co. Wicklow, Ireland), available on the website for raw data processing.

#### 2.4.2. ATR-FTIR Analysis

The FTIR spectra of milk/ME/GPE lyophilized powder and control TM sample were recorded by an ${IRAffinity}^{-1}$ spectrometer equipped with an ATR unit (Shimadzu, Kyoto, Japan). The spectra were collected in the wavenumber range of 4000–600 ${cm}^{-1}$, from 100 scan accumulations and 4 cm^−1^ resolution.

#### 2.4.3. DLS Measurements

Particle size of optimized lyophilized milk/ME/GPE powder and control TM sample (spray-dried thermally treated goat milk) were determined by dynamic light scattering (DLS), using a NanoPartica SZ-100 device (Horiba Ltd., Kyoto, Japan). Before analysis, both powders were reconstituted in MilliQ water to prepare 0.1% solutions. The measurements were conducted at 25 °C, in polydisperse mode, in five replicates. Particle size distributions are reported as intensity-weighted distributions.

#### 2.4.4. Electrophoretic Analysis

SDS-PAGE in reducing conditions (SDS-R-PAGE) was used for protein profile characterization of optimized milk/ME/GPE powder and control TM sample, as previously described in detail [30,31]. SDS-R-PAGE was performed using separating (12.5% *w*/*v*; pH = 8.85) and stacking gels (5% *w*/*v*; pH = 6.8), as well as Tris-Glycine running buffer (0.05 M Tris (pH = 8.5), 0.19 M glycine, 0.1% *w*/*v* SDS). The sample was prepared by dissolving 4 mg of lyophilized milk/ME/GPE powder and the control TM sample in SDS-R-PAGE sample buffer, which consisted of 0.055 M Tris-HCl (pH = 6.8), 2% (*w*/*v*) SDS, 7% (*v*/*v*) glycerol, 0.0025% (*w*/*v*) bromophenol blue, and 5% β-mercaptoethanol. For all electrophoretic techniques, aliquots of 25 μL were loaded into the wells. After analysis, gels were stained with Coomassie blue dye for 1 h, then destained, scanned, and analyzed using SigmaGel software (SigmaGel software version 1.1, Jandal Scientific, San Rafael, CA, USA).

#### 2.4.5. SEM Microscopy of Functional Ingredient

The morphology of optimized milk/ME/GPE lyophilized powder and control TM sample was recorded by Scanning Electron Microscopy (JEOL JSM-6390LV, Tokyo, Japan) at an acceleration voltage of 30 kV. Before imaging, the powders were attached to metallic stubs and coated with a layer of gold using sputter-coating for 100 s, at 30 mA, within a BALTEC SCD 005 sputtering chamber (New York, NY, USA).

#### 2.4.6. UHPLC-Q-ToF-MS Analysis

Lyophilized milk/ME/GPE powder was mixed with 80% methanol containing 0.1% HCl (1:10, *w*/*v*) and stirred for 1 h using a mechanical shaker. The mixture was then centrifuged at 17,000× *g* for 10 min. The resulting supernatant was filtered through a 0.22 µm membrane filter and analyzed by UHPLC–Q-TOF-MS (Agilent 1290 Infinity ultra-high-performance liquid chromatography system coupled with a quadrupole time-of-flight mass spectrometer, 6530C Q-TOF-MS; Agilent Technologies, Inc., Santa Clara, CA, USA), following the method previously described by Milinčić et al. [32]. Chromatographic separation was performed at 40 °C on a Zorbax C_18_ column (2.1 × 50 mm, 1.8 µm; Agilent Technologies, Inc., CA, USA). The mobile phase consisted of: (A) ultrapure water and (B) 98% acetonitrile (MS grade), both containing 0.1% HCOOH (MS grade). The flow rate was constant at 0.3 mL min^−1^, and the injection volume was 5 µL. The gradient elution program started at 2% solvent B and was held for 2 min, then increased to 98% B over the next 17 min. Subsequently, the gradient was returned to the initial conditions (2% B) over 5 min to re-equilibrate the column. The Q-ToF MS system was equipped with a Dual Agilent Jet Stream electrospray ionization (ESI) source, operating in both positive (ESI+) and negative (ESI−) ionization modes. The ESI operating parameters were set as previously reported by Milinčić et al. [32]. Data-dependent acquisition (DDA) was employed for suspect screening using the Auto MS/MS acquisition mode (*m*/*z* 100–1700, scan rate 1 spectrum s^−1^) with a collision energy of 30 eV. Agilent MassHunter software (MassHunter Qualitative Analysis, version 10.0; Agilent Technologies, Inc., CA, USA), in combination with MS-DIAL software (version 4.60; RIKEN, Yokohama, Kanagawa, Japan http://prime.psc.riken.jp/), was used for screening, extraction of relevant data (retention time, monoisotopic mass and peak area) and the subsequent evaluation, analysis and presentation of MS data. Bioactive compounds were identified based on monoisotopic mass and MS fragmentation patterns, in accordance with previously reported literature data [9,29,33,34,35]. Identified phenolic compounds were quantified using available standards and expressed in µg/g of lyophilized powder. In the absence of authentic standards, some tentatively identified phenolic compounds were semi-quantified using structurally similar standards and expressed as equivalents of the corresponding reference compounds. Table S3 presents the regression equations and associated parameters for the standards used for the quantification or semi-quantification of (tentatively) identified phenolic compounds in the methanolic extract of the optimized milk/ME/GPE powder.

#### 2.4.7. Techno-Functional Properties of Functional Ingredient

Emulsifying properties, determination of emulsion activity index (EAI) and emulsion stability index (ESI), as well as foaming properties, were described in our previous study [5].

Water/Oil holding capacities (WHC/OHC) were determined according to Wani et al. [36], with slight modification. Briefly, 1 g of the sample was mixed with 10 mL of deionized water/oil in a centrifuge tube and then stirred for one minute. The sample was allowed to stand for 30 min at room temperature and centrifuged at 2000× *g* for 30 min. The quantity of obtained supernatant was measured. Water/oil absorption capacity was calculated as the difference between the initial volume of water/oil and the volume of the supernatant expressed as grams of water/oil absorbed per 100 g of dry sample.

### 2.5. Physicochemical Characterization of Fortified Soup

The moisture content was determined using the standard gravimetric method by drying at 105 °C until a constant weight [37].

Water solubility of dehydrated soups was determined according to Belščak-Cvitanović et al. [38], with slight modification. The 0.1 g of dehydrated soups was suspended in 1 g of distilled water at 30 °C. The suspension was stirred for 30 min and then centrifuged at 9500 rpm for 10 min. The supernatant was decanted into a beaker and dried at 105 °C to constant mass. The solubility of the dehydrated soups was determined from the mass obtained after drying and the initial mass of the sample. The solubility was expressed as g/100 g of dry sample.

Wettability was determined according to Belščak-Cvitanović et al. [38].

The bulk density (ρB) was calculated as the ratio between the mass of the dehydrated soups and the volume they occupy in the beaker. The tapped density (ρT) was calculated as the ratio between the mass of the dehydrated soups and their volume after 500 taps [39].

The compressibility index (CI) and Hausner ratio (HR) were calculated using the following equations:

(1) $$CI\left(\%\right)=\frac{\rho T-\rho B}{\rho T}\cdot 100$$

(2) $$HR=\frac{\rho T}{\rho B}$$

### 2.6. In Vitro Gastrointestinal Digestion of Functional Ingredient

The bioaccessibility of phenolic compounds after simulated gastrointestinal digestion was evaluated using a static in vitro digestion model adapted from the protocol described by Aura et al. [40]. Briefly, 1 g of functional ingredient was dispersed in 15 mL of Milli-Q water and 10 mL of 0.85% NaCl solution. Salivary amylase (50 U) was added, the pH was adjusted to 6.9 and the mixtures were incubated at 37 °C for 5 min under continuous shaking (120 rpm) to simulate the oral phase. The resulting oral bolus was then combined with 4.5 mL of 150 mM HCl and 1 mL of pepsin solution (2 mg/mL). The pH was adjusted to 2.5, and samples were incubated at 37 °C for 2 h with continuous shaking to simulate gastric digestion. For the intestinal phase, gastric chyme was supplemented with 4 mL of bile acid solution (deoxycholic and cholic acids), 4 mL of pancreatin solution (18.75 mg/mL) and 1 mL of mucin solution. The pH was adjusted to 7.0, and the mixtures were further incubated at 37 °C for 3 h. After digestion, Milli-Q water was added to obtain a final volume of 45 mL. The digested sample was centrifuged, and the resulting supernatant was collected. Before chromatographic analysis, collected supernatant was subjected to solid-phase extraction using SPE (CLEAN-UP^R^, C18 extraction columns, unendcapped-PKG50, UCT, Bristol, UK), to remove impurities and concentrate phenolic compounds. The retained phenolics were then recovered by elution with 1 mL of methanol containing 0.1% HCl. Functional ingredient (1 g) extracted with 45 mL of methanol containing 0.1% HCl, without digestion, was used as a control.

Separation, identification and quantification/semi-quantification of selected phenolic compounds before and after digestion were performed using UHPLC Q-ToF MS analysis as previously described in Section 2.4.6. The bioaccessibility of the individual and total identified phenolic compounds (BI%) was calculated according to the following Equation (3):

(3) $$Bioaccessibility\left(BI\right),\%=\frac{\sum PCd}{\sum PCc}\times 100$$

where PCd is the content (µg/g of initial powder) of each identified phenolic compound in the digested samples, and PCc is the content (µg/g of initial powder) of each identified phenolic compound in the control sample.

### 2.7. Sensory Analysis and Antioxidant Properties

#### 2.7.1. Preparation of Dehydrated Soups for Sensory Analysis

For the examination of the properties of optimized ingredient within a food product, optimized functional ingredient was incorporated into a model-dehydrated soup (Regulation on the Quality of Soups, Sauces, Meal Additives, and Related Products, Official Gazette of FRY, 41/93, and Official Gazette of SCG, 56/2003 and 4/2004). The ingredients for the soup were as follows: garlic powder (Spice Chef, Maxi, Belgrade, Serbia); onion powder (Premia, Maxi, Serbia); black pepper (Začin C, Nestlé, Belgrade, Serbia), sea salt (Kristal So doo, Belgrade, Serbia); starch (Dr. Oetker, Belgrade, Serbia), without vegetable oil [41]. The composition of dehydrated soup (S) was as follows: 12.25% garlic powder, 12.25% onion powder, 10.5% black pepper, 11.9% sea salt, 30% starch and 17.5% functional milk/ME/GPE ingredient.

The control soup was composed of the following ingredients (C): 12.25% garlic powder, 12.25% onion powder, 10.5% black pepper, 11.9% sea salt, 35.6% starch and 17.5% thermally treated defatted goat milk. The commercial soup (CS) was Quik e-free instant fat-free vegetable soup, Aleva d.o.o., Novi Kneževac, Serbia.

The composition of the commercial instant soup, according to its declaration, was as follows: maltodextrin, table salt 18%, dried vegetables up to 14% (carrot, onion, parsnip, tomato, leek, celery), modified corn starch, dextrose, flavor enhancers: monosodium glutamate 5.5%, disodium 5′-ribonucleotides, hydrolyzed soy proteins, yeast extract, spices, soy sauce, vegetable extract, sugar, rapeseed oil and flavors. Energy value: 90 kJ (21.5 kcal) per 10 g of product; average nutritional value: proteins 0.65 g/10 g; carbohydrates 5.4 g/10 g; fats 0.022 g/10 g.

The solid ingredients were mixed in the given proportions for soups S and C, while the commercial soup was prepared according to the instructions. The samples S and C were dissolved in water at a 1:20 *w*/*v* ratio and then subjected to cooking at 95 °C for 5 min, following Sugumar and Guha [41].

#### 2.7.2. Sensory Properties

The sensory analysis of the soup was conducted by 10 certified evaluators, using both the “overall quality evaluation” and the “consumer acceptance evaluation”, with 28 evaluators (17 women, 11 men) who were potential consumers. Sensory analyses were conducted in accordance with the Code of Professional Ethics of the University of Belgrade [42]. These questionnaires, prepared for sensory analysis, are in line with the General Regulation on Data Protection of the European Union. According to the relevant regulations and institutional policies, ethics approval was not required. All participants provided written informed consent prior to participation. At the beginning of the sensory examination, all panelists gave written consent to participate, and they were aware they could withdraw from the study at any time, that their responses were confidential, that the responses would be used for scientific purposes, as well as that the participants’ data and their answers would not be published without their knowledge. Before sensory evaluation, participants were fully informed about study requirements. The tested samples were safe for consumption.

Soup samples were prepared for sensory analysis as previously described in Section 2.7.1. and were placed in glass cups. The soups were prepared immediately before the sensory analysis. The soup samples were heated to a temperature of 40 °C ± 2 °C. Each sample was marked with a one-digit number. Tasting was conducted with plastic spoons.

##### Overall Quality Evaluation

Sensory analysis by overall quality evaluation was carried out by point system testing on a scale range from 0 to 5: excellent quality (quality score > 4.5), very good quality (3.5 < score ≤ 4.5), good quality (2.5 < score ≤ 3.5), poor/unsatisfactory quality (1.5 < score ≤ 2.5), very poor quality (score ≤ 1.5) [43]. The panelists were provided with the evaluation chart with scale range and were asked to score the parameters: taste, color, smell, texture and overall acceptability. With the fact that they should have evaluated the taste parameter in particular: salty, sweet, bitter, sour and the presence of umami feeling.

##### Consumer Acceptance Evaluation

Sensory analysis was carried out by consumer acceptance evaluation on a preferential scale range from 1 to 9 where 1 = extremely dislike, 2 = very much dislike, 3 = moderately dislike, 4 = slightly dislike, 5 = neither like/nor dislike, 6 = slightly like, 7 = moderately like, 8 = very much like, 9 = extremely like [43]. The panelists were provided with the evaluation chart with scale range and were asked to score the parameters: taste, color, smell, texture, mouthfeel and overall acceptability.

#### 2.7.3. Antioxidant Properties of Fortified Soup Before and After Simulated Gastrointestinal Digestion

Five tests already used for optimization of the milk/ME/GPE functional ingredient were applied for determination of antioxidant properties of dehydrated soups before and after in vitro gastrointestinal digestion as previously described in Section 2.3.2. A 1 g sample of soup powder was subjected to in vitro gastrointestinal digestion, whereas an equivalent amount of soup powder (1 g) reconstituted in 45 mL of milli-Q water served as the control sample. Following digestion, both the digested and control soup suspensions were centrifuged at 4000× *g* for 10 min. The resulting supernatants were collected and subjected to further analysis.

### 2.8. Statistical Analysis

All analyses related to the optimized milk/ME/GPE ingredient were carried out in triplicate and the results are presented as mean values ± standard deviation. For the optimization studies, analysis of variance (ANOVA) associated with the response surface methodology was applied to evaluate the significance of the investigated factors and the adequacy of the developed models. Differences between two groups were tested for statistical significance using Student’s *t*-test at *p* < 0.05 (StatSoft Co., Tulsa, OK, USA). Prior to the application of parametric tests, normality and homogeneity of variance were assessed. This approach was applied to the emulsion stability index (ESI), the physicochemical and antioxidant properties of the control and functional soups. Sensory evaluation data were analyzed using the non-parametric Kruskal–Wallis test (*p* < 0.05), followed by Dunn’s post hoc test to determine differences among soup samples for each sensory attribute. Data presented in radar diagrams are expressed as mean values. All graphs were generated using GraphPad Prism 6 software (San Diego, CA, USA).

## 3. Results and Discussion

### 3.1. Design of Experiment—DoE and Central Composite Design—CCD

The concept of surface methodology (RSM) has already been described in Section 2.3, of the Materials and Methods Section. The basic idea of RSM is that, based on a well-designed experimental plan (such as a central composite design), it is possible to obtain a dataset suitable for the development of an empirical model (usually a second-order polynomial equation) (Equation (1)) that describes how changes in the factors affect the response [44]. Once the model is established, response surfaces (three-dimensional surface plots or contour plots) can be constructed to visually illustrate the relationship between the factors and the response, making it easier to identify optimal conditions [45].

(4) $$y=\beta_{0}+\sum_{i=1}^{k}\beta_{i}x_{i}+\sum_{i=1}^{k}\beta_{ii}{x_{i}}^{2}+\sum_{1\leq i\leq j}^{k}\beta_{ij}x_{i}x_{j}+\varepsilon$$

where *y* represents the response variable; $x_{i}$ and $x_{j}$ are independent variables (*i* and *j* can range from 1 to k); $\beta_{0}$ is the intercept coefficient; $\beta_{i},\beta_{ii}\;and\;\beta_{ij}$ represent coefficients of linear, quadratic, and interaction effects, respectively; *k* is the number of independent parameters (k = 2 in this study), and *ε* is the residual error [46]. Statistical analysis was performed on the experimental results, and 95% confidence intervals were determined for each factor individually as well as for combinations of factors.

Five antioxidant responses: total phenolic content (TPC), ABTS*+ and DPPH* radical scavenging activity, ferric reducing power (FRP) and ferrous chelating ability (CHE), were evaluated as a function of the content of the grape pomace seed and mushroom extracts in the goat-milk based functional ingredient. The relative magnitudes of the effects of investigated variables were determined based on the factor effects and their corresponding *p*-values.

**Figure 1:**
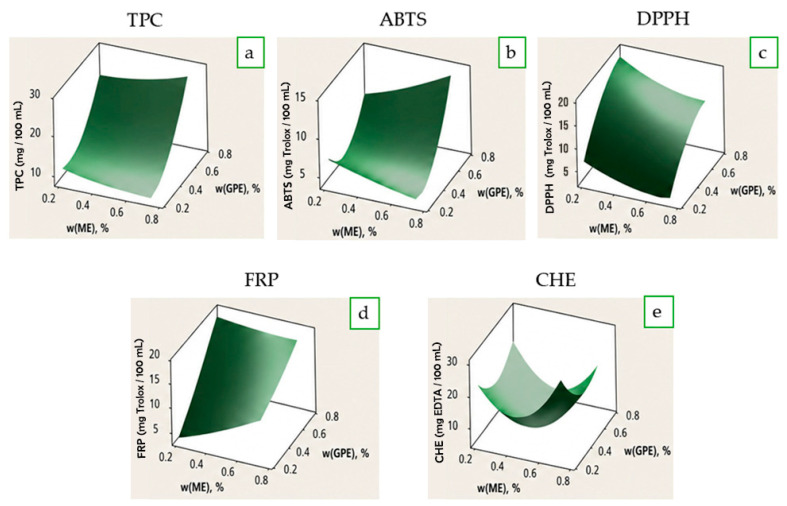
Response surface plot for (**a**) total phenolic content—TPC; (**b**) ABTS*+ scavenging activity-ABTS; (**c**) DPPH* scavenging activity-DPPH; (**d**) ferric reducing power—FRP; and (**e**) ferrous chelating ability—CHE.

The estimated effects and regression coefficients for TPC are presented in Table S4 together with the analysis of variance (ANOVA), which was used to evaluate the influence of the investigated factors on the total phenolic content. By comparing the estimated *p*-values with the significance threshold (*p* ≤ 0.05), it was found that the linear term of grape pomace seed extract significantly influences the TPC in the analyzed mixtures (Figure 1a). The obtained coefficient of determination ($R^{2}$ = 69.93%) indicates a moderate level of correlation between the response variable and the independent factors. Furthermore, the lack of fit test (*p* > 0.05) suggested that the model adequately described the experimental data within the investigated design space.

The data listed in Table S5 were used to evaluate the effect of mushroom and grape pomace seed extracts on ABTS*+ scavenging activity of the prepared mixtures. The results suggested that the content of grape pomace seed extract was the only statistically significant factor affecting the response. The coefficient of determination was $R^{2}$ = 67.47%, indicating a moderate level of correlation between the response variable and independent factors. The significance of linear regression was confirmed by the analysis of variance (*p* ≤ 0.05). The response surface plot (Figure 1b) clearly shows a linear increase in antioxidant activity with increase in grape pomace seed extract. Although the quadratic term of *w*(GPE) was not statistically significant, its *p*-value close to the significance threshold (*p* = 0.05) suggests the presence of slight curvature in the response surface, which is also visible in the contour plot (Figure 1b).

Table S6 summarizes the estimated regression coefficients and the analysis of variance for the DPPH* scavenging activity. Similar to TPC and ABTS*+ scavenging activity responses, the antioxidant activity analyzed by the DPPH* assay was significantly influenced by the linear term of GPE content.

The determination coefficient ($R^{2}$ = 78.74%) indicates a relatively good correlation between the response variable and the independent factors, suggesting acceptable model fitting. The linear trend is also evident from the response surface plot (Figure 1c), where DPPH* scavenging activity increases with increasing GPE content and reaches a maximum at *w*(GPE) 0.50% (*m*/*m*). Furthermore, the response surface indicates that increasing the portion of mushroom extract, leads to a decrease in DPPH* antioxidant activity. The influence of the investigated parameters on antioxidant activity measured by the FRP assay was evaluated based on the corresponding *p*-values (Table S7). The obtained coefficient of determination (*R*^2^ = 57.29%) indicates a low level of correlation, suggesting that the model has limited predictive ability for this response. In addition, the presence of significant lack of fit further confirms that the model does not adequately describe the experimental data. Despite this, the linear term of GPE content showed a statistically significant effect on ferric reducing power. The response surface plot (Figure 1d) indicates that FRP increases with increasing GPE content, reaching its maximum at the highest investigated level of this factor.

The estimated regression coefficients and analysis of variance results presented in Table S8 indicate that none of the investigated factors had a statistically significant effect on ferrous chelating ability and the investigated parameters. Accordingly, no meaningful correlation between the response and the independent variables was observed. The response surface plot (Figure 1e) shows that the lowest chelating ability was observed at the intermediate levels of both extracts, although this trend cannot be considered statistically reliable due to the lack of significant model terms.

### 3.2. Overall Optimization—Desirability Function

Due to the obtained results, three response variables: TPC, ABTS*+ and DPPH* radical scavenging activities, were selected for simultaneous optimization using the desirable function approach. This approach was applied to determine the optimal levels of the investigated factors that simultaneously satisfy multiple response criteria [47]. The regression equations, together with the corresponding coefficients of determinations, are summarized in Table S9.

To calculate the overall desirability, the relative importance of all three responses (TPC, ABTS*+ and DPPH*) was set equally, with an importance value of one assigned to each response. For TPC and ABTS*+ scavenging activity, the goal was set to maximize the response, as higher values indicate improved antioxidant properties. For DPPH scavenging activity, a minimization criterion was applied during multi-response optimization in order to obtain the highest overall desirability of the system. The optimization procedure aimed to identify a balanced compromise among all responses rather than maximize each response individually [48]. Greater weight was given to TPC and ABTS*+ scavenging activity responses because ABTS*+ scavenging activity is considered more suitable for evaluating antioxidant capacity in complex food matrices containing both hydrophilic and lipophilic compounds [49]. The target lower and upper limits for each response were defined based on the experimentally obtained values (Table S10). The overall desirability was calculated as the geometric mean of individual desirability functions. Finally, the recovery was calculated as the ratio of the experimentally obtained and predicted values.

According to the established dataset, overall desirability obtained for the optimized formulation was *D* = 0.9377, corresponding to a mixture containing 0.50% (*m*/*m*) of both mushroom and grape pomace seed extracts.

The predicted and experimentally obtained values of the responses are presented in Table 1.

A relatively good agreement between predicted and experimental values was observed for total phenolic content. However, notable deviations were recorded for antioxidant activity measured by the ABTS*+ and DPPH* assays. Specifically, the experimental value for the DPPH* assay was considerably higher than the predicted value, while the experimental ABTS*+ value was substantially lower than predicted.

Such discrepancies between predicted and experimental values are not uncommon in response surface methodology when regression models exhibit moderate coefficients of determination. In the present study, the obtained R^2^ values ranged from 67.47 to 78.74%, indicating that the models adequately describe the general trends of the responses but possess limited predictive accuracy for precise estimation of the optimal values. Similar deviations between predicted and experimental responses have been reported in several RSM optimization studies dealing with phenolic compounds and antioxidant activity [5,50]. Sady, Matuszak and Blaszczyk [50] reported that deviations exceeding 30–40% may occur when model validation is performed near or beyond the boundaries of the experimental design space. Additionally, Milinčić et al. [5] observed discrepancies between predicted and experimental antioxidant responses, attributing them to the differences in assay mechanisms and complexity of food matrices. Furthermore, the polynomial models obtained in the present study were predominantly linear, indicating that the responses were surfaces mainly governed by the concentration of grape pomace seed extract. Such models may not fully capture potential curvature or interaction effects, which can contribute to deviations between predicted and experimental values during validation. Therefore, the developed CCD model should be considered primarily as a screening and formulation optimization tool that identifies general trends and promising formulation regions rather than providing highly accurate quantitative prediction of antioxidant responses.

However, CCD was successfully applied to optimize a goat milk-based functional ingredient enriched with mushroom extract, resulting in enhanced phenolic content and antioxidant activity [5]. Ebrahimi et al. [51] applied a CCD to optimize conditions for ultrasound-assisted extraction of polyphenols from sugar beet leaves, obtaining an extract with a high content of phenolic compounds and improved antioxidant efficacy.

### 3.3. Physicochemical Characterization of Functional Ingredient

#### 3.3.1. Glucan Content in Optimized Milk/ME/GPE Powder

Total glucan content was 25.43 ± 0.18% (*w*/*w*), while individual contents of α- and β-glucan were 1.85 ± 0.08% (*w*/*w*) and 23.58 ± 0.09% (*w*/*w*), respectively. In our previous study, the ME/M ingredient (goat milk enriched with mushroom extract) contained 26.62% total glucans, 1.94% α-glucans, and 24.67% β-glucans [5]. This confirms that the incorporation of mushroom extract is the primary contributor to the glucan content of the formulated ingredient.

#### 3.3.2. ATR-FTIR Analysis of Optimized Milk/ME/GPE Powder

**Figure 2:**
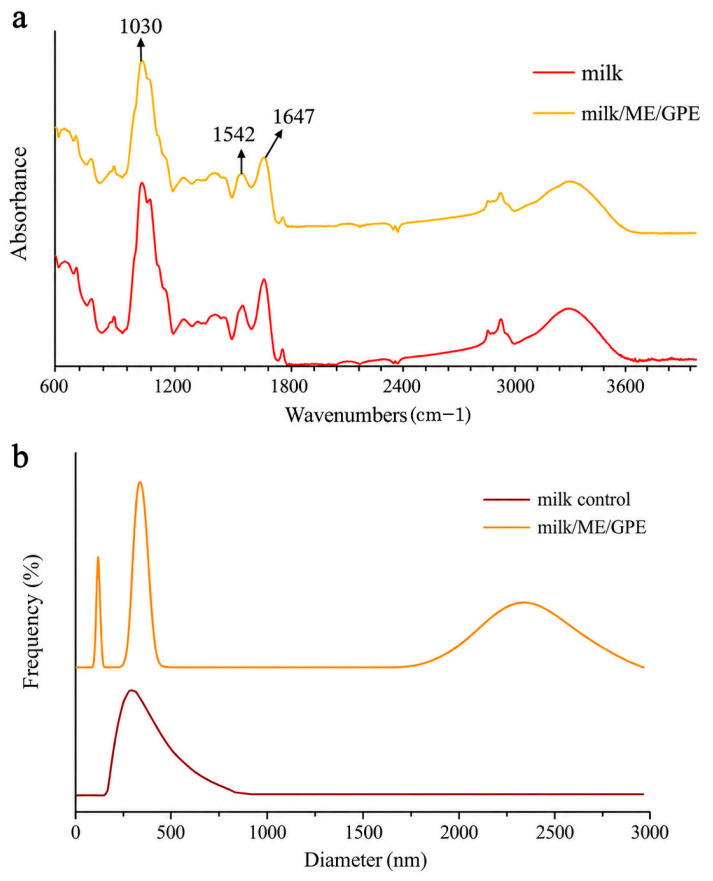
(**a**) ATR-FTIR spectra of the optimized milk/ME/GPE ingredient and control milk (TM) powder. Abbreviations: milk/ME/GPE—thermally treated milk/mushroom extract/grape pomace seed extract. TM—thermally treated skimmed goat milk; (**b**) particle size distribution of the optimized milk/ME/GPE powder and control TM sample.

Samples were analyzed by ATR-FTIR spectroscopy to evaluate potential changes in the chemical composition arising from interactions between bioactive compounds from mushroom and grape pomace seed extracts and milk compounds, as well as to assess the structural stability of the obtained functional ingredient. As shown in Figure 2a, the ATR-FTIR spectra of milk/ME/GPE functional ingredient are highly similar to those of the control sample (TM). The spectra are dominated by bands characteristic of major milk compounds, primarily from lactose (around 1030 ${cm}^{-1}$) and proteins (amid I and II bands at approximately 1647 ${cm}^{-1}$ and 1542 ${cm}^{-1}$, respectively). This indicates that the overall spectral profile is governed by the dominant milk matrix. No new distinct bands were observed that could be attributed to proteolysis of proteins or the presence of phenolic–protein interactions. However, it is important to emphasize that the absence of new bands in ATR-FTIR spectra does not necessarily indicate the absence of molecular interactions. In complex multicomponent systems, such as milk-based matrices enriched with plant extracts, spectral contributions of minor components are often masked by the dominant signals of proteins and lactose, particularly in the fingerprint region. A similar behavior was observed in goat’s milk powder enriched with goji berry extracts, where ATR-FTIR spectra were dominated by milk components [52]. However, Minić et al. [23] reported interactions between mushroom polysaccharides and milk proteins, while Milinčić et al. [53] observed interactions between phenolic compounds from grape pomace seed extract and milk proteins. In both cases, these interactions were investigated using a combination of spectroscopic techniques, including ATR-FTIR and Raman spectroscopy, coupled with advanced chemometric analysis, which enabled the detection of subtle structural modifications that were not clearly resolved by conventional ATR-FTIR analysis alone. It should also be noted that these studies were performed using systems containing a single type of extract, whereas the present study involves a more complex formulation with both mushroom and grape pomace seed extracts incorporated into a milk matrix. Such increased system complexity may further contribute to overlapping spectral features and reduced detectability of interactions using ATR-FTIR spectroscopy.

Therefore, the absence of pronounced spectral changes in the present study may be attributed to a combination of factors, including the multicomponent nature of the system, the relative proportion of added extracts and the limitations of ATR-FTIR spectroscopy in resolving subtle molecular interactions under these conditions. Based on the obtained results, it can be concluded that the applied formulation leads to the formation of a homogeneous and structurally stable powder.

#### 3.3.3. Dynamic Light Scattering (DLS) Measurements of Optimized Milk/ME/GPE Powder

Although ATR-FTIR spectroscopy provides valuable information on the overall chemical fingerprint of the milk/ME/GPE powder, this technique has inherent limitations when it comes to resolving subtle structural and conformational changes in complex food matrices. Broad and overlapping bands in the amide and carbohydrate regions, together with the relatively low proportion of added extracts compared to the bulk milk phase, can mask fine spectral differences associated with protein–polyphenol interactions, partial proteolysis or changes in a complex food system [54,55]. For this reason, ATR-FTIR data are best interpreted in conjunction with complementary physicochemical methods that can probe particle size distribution and protein molecular profiles more directly [5,23,56]. The obtained ATR-FTIR (Figure 2a) and DLS (Figure 2b) results for the control milk powder (TM) are fully consistent with the previously published data reported by [5], indicating a relatively homogeneous dispersion of particles (primarily casein micelles). This result is consistent with literature values for heated milk powders, where casein micelles remain intact and uniform in size [23,53]. In contrast, the milk/ME/GPE sample showed a polymodal particle size distribution and high polydispersity, with low reproducibility, suggesting the presence of several particle populations and a more complex colloidal organization. The enriched sample is heterogeneous, containing casein micelles (~290 nm) along with smaller nanoparticles (~100 nm) and much larger aggregates (~2.2 μm). The DLS analysis reveals that the addition of ME and GPE to milk altered the colloidal size distribution of the system, in agreement with recent literature findings. The observed polymodal distribution may be associated with several phenomena previously reported in similar systems, including protein modification, polyphenol–protein interactions, and polysaccharide–protein interactions [5,23,57,58]. The presence of GPE (rich in polyphenols) may have contributed to the formation of larger aggregates, potentially through protein–polyphenol interactions [58]. In addition, mushroom-derived components, including proteolytic enzymes and β-glucans, may have influenced protein modification and intermolecular interactions, leading to a broader particle size distribution [5].

#### 3.3.4. Electrophoretic Analysis of Optimized Milk/ME/GPE Powder

**Figure 3:**
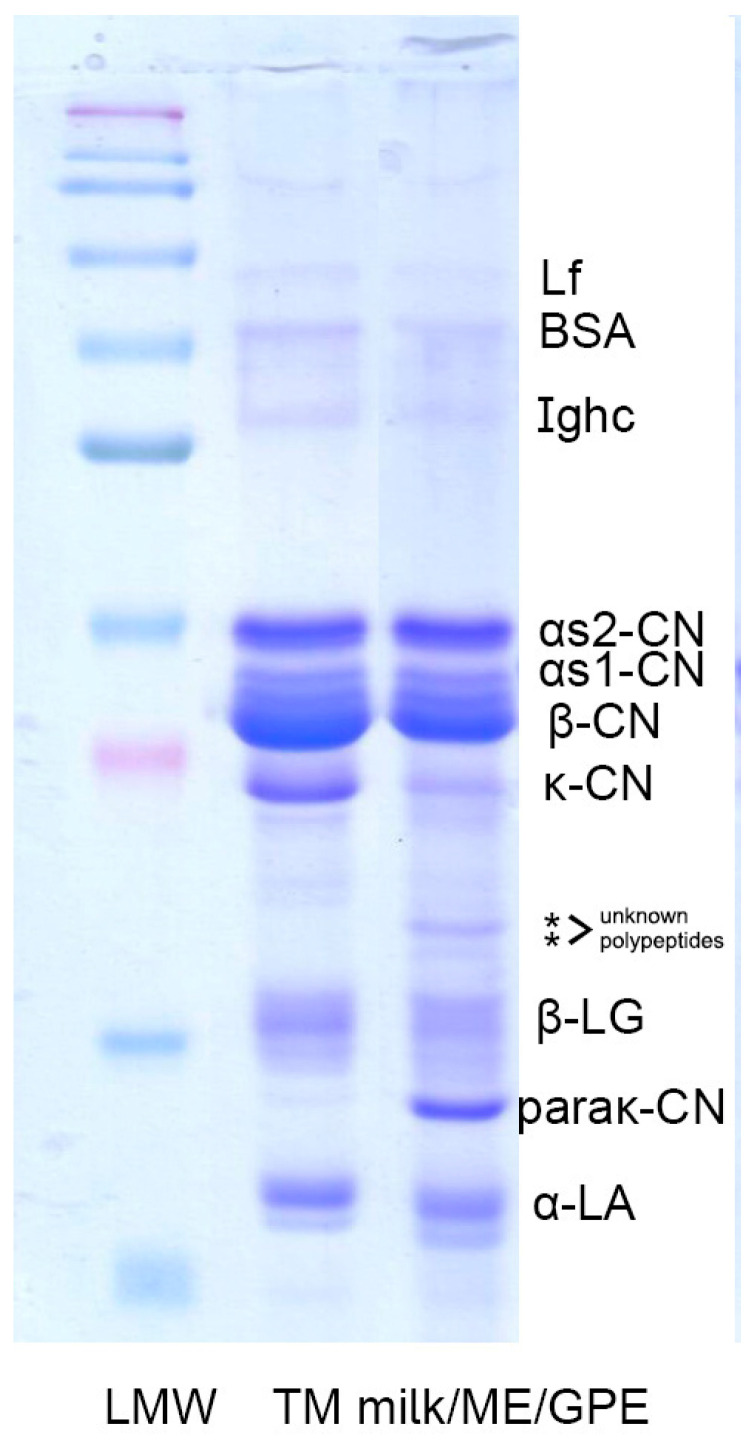
Electrophoretic patterns of the optimized milk/ME/GPE and control TM powders, analyzed by SDS-PAGE in reducing conditions (SDS-R-PAGE). Bovine casein standard (ST-BCN), milk/ME/GPE—thermally treated milk/mushroom extract/grape pomace seed extract. TM—thermally treated skimmed goat milk. Abbreviations: Lactoferrin (Lf); bovine serum albumin (BSA); Imunoglobulin heavy chain (Ighc); αs2-casein (αs2-CN); αs1-casein (αs1-CN); β-casein (β-CN); κ-casein (κ-CN); β-lactoglobulins (β-LG); paraκ-casein (paraκ-CN); α-lactalbumins (α-LA). * low-molecular-weight polypeptides.

Protein profiles of milk/ME/GPE and control TM samples were analyzed using SDS-PAGE under reducing conditions (Figure 3). The identification of proteins was performed using the standard of bovine milk caseins and previously published literature data for goat milk samples [23,30,31].

As can be seen, well-known protein bands of goat’s milk, corresponding to caseins and whey proteins, can be observed on the control TM pattern (milk TM (C)). On the other hand, well-known protein bands (caseins and whey proteins), paraκ-casein and two new polypeptide bands (labeled with *, Figure 3, milk/ME/GPE pattern) can be observed on the milk/ME/GPE pattern. These non-characteristic polypeptide bands were not detected on the control TM pattern, but they have the same electrophoretic pathways as the bands detected in a previous study on the milk/ME pattern [5]. Thus, the SDS-PAGE analysis revealed noticeable changes in the protein profile of the milk/ME/GPE functional ingredient compared to the control sample. The polypeptide composition of the dominant proteins of optimized milk/ME/GPE and control TM powders is presented in Table S11. The appearance of paraκ-casein and lower-molecular-weight bands and reduced relative abundance of higher-molecular-weight fractions (β-casein, κ-casein and αS1-casein), is consistent with partial protein hydrolysis, particularly of κ-casein, as also indicated by densitometric profiles (Figure S1). The observed protein profile may be associated with the proteolytic activity previously reported for edible mushrooms [23], although other mechanisms, including protein–polyphenol interactions, may have also contributed to the observed changes.

Milinčić et al. [31] reported that, although all expected casein and whey protein bands were present under reducing SDS-PAGE conditions, their staining intensities decreased with increasing levels of grape pomace seed extract. This effect was attributed to interactions between polyphenols and milk proteins, which reduced the amount of extractable protein detected on the gel. Similar protein–polyphenol interactions have been reported by Mudgil et al. [59], who observed changes in SDS-PAGE band intensity, due to complex formation between date seed polyphenols with camel milk proteins. Lima et al. [60] reported that phenolic compounds can modulate proteolysis by interacting with milk proteins or enzymes, thereby affecting the degradation of caseins. Wang et al. [61] found that a cysteine peptidase from *Moringa oleifera* showed preferential cleavage of κ-casein, resulting in the formation of lower-molecular-weight peptides detectable by SDS-PAGE.

#### 3.3.5. SEM Microscopy of Optimized Milk/ME/GPE Powder

**Figure 4:**
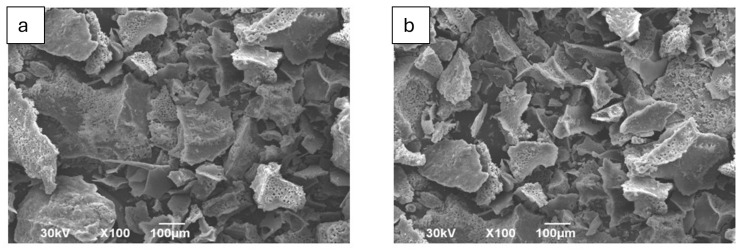
SEM images of optimized milk/ME/GPE lyophilized powder obtained from two different scans (**a**,**b**). Abbreviations: milk/ME/GPE—thermally treated milk/mushroom extract/grape pomace seed extract.

Scanning electron microscopy (SEM) revealed the morphological characteristics of optimized milk/ME/GPE lyophilized powders (Figure 4).

As can be observed in the SEM images, the particles of the milk/ME/GPE sample exhibit irregular structures, resembling rough, torn flakes or broken porous glass (Figure 4a,b) [62]. These torn flakes exhibit a rugged surface with visible tiny voids (honeycomb-like structure) (Figure 4a,b), which were probably formed as a consequence of ice crystal sublimation [5,23], or may also be associated with protein structural modifications suggested by electrophoretic analysis (Figure 3, milk/ME/GPE pattern).

#### 3.3.6. UHPLC Q-ToF MS Analysis of Optimized Milk/ME/GPE Powder

The untargeted analysis was applied to identify all grape- and mushroom-derived bioactive compounds present in the milk/GPE/ME powder (Table 2). In this case, all bioactive compounds extracted from milk/GPE/ME powder were identified, including free bioactive compounds and phenolics weakly bound to the surface of casein micelles [31]. A total of 44 compounds were identified, including phenolic acids (16 compounds), flavonoids (24 compounds), amino acids and fatty acids (4 compounds). All the tentatively identified phenolic compounds most likely originate from grape seed [9,29,34,35], while the detected fatty acids and phenylalanine are characteristic of mushroom-derived components [28,33,63]. As phenolic compounds were the most frequently detected, they were quantified or semi-quantified using available standards to additionally determine their individual and total contents in milk/GPE/ME powder (Table 2). Phenolic acids were the most abundant, with a total content of 153.32 µg/g LP, followed by procyanidins (37.99 µg/g LP) and flavan-3-ols (23.50 µg/g LP), while other detected phenolic compounds were mostly present in trace amounts (<LOQ). Among detected phenolic acids, gallic acid (**3**) and its derivatives were the most numerous and were mainly identified in the form of glycosides (**9**, **10**, **13** and **14**), esters with shikimic acid (**8**), and methyl/ethyl derivatives (**4** and **5**). These compounds exhibit unique monoisotopic masses and common gallic acid–derived fragments, such as fragments at 169 *m*/*z* (Y_0_^–^ ion; deprotonated gallic acid), 168 *m*/*z* (radical anion, [Y_0_–H]^–^) and 125/124 *m*/*z* (–CO_2_). Ellagic acid (a dimeric derivative of gallic acid; **6**) and its conjugates, such as galloyl-HHDP-hexose (**16**) and ellagic acid pentoside (**12**), were also detected. Key fragments for the identification of galloyl-HHDP-hexose were obtained through the sequential loss of a galloyl moiety and hexosyl unit [*m*/*z* 633 → 463 *m*/*z* [–Gal; –H_2_O] → 301 *m*/*z* [–Hex]). Moreover, gallic and ellagic acid were additionally confirmed by direct comparison with available standards. Compounds **7** and **15** were identified as vanilloside and dicaffeoylquinic acid, respectively, but were present only in trace amounts. Hydroxybenzoic acid (**1**) and dihydroxybenzoic acid (**2**) were also detected; however, their presence in milk/GPE/ME powder may also originate from the mushroom extract, considering that these compounds have previously been reported in *Agrocybe aegerita* extracts [28]. Quantification of individual phenolic acids revealed the highest contents of ethyl gallate (54.05 µg GA/g LP) and gallic acid (47.78 µg/g LP), followed by dihydroxybenzoic acid (19.24 µg GA/g LP) and ellagic acid (11.70 µg/g LP).

Flavan-3-ols and procyanidins are typical grape seed-derived compounds, which explains their presence in milk/GPE/ME powder. Among monomeric flavan-3-ols, catechin was predominant (13.36 µg/g LP), followed by epicatechin (5.08 µg/g LP) and catechin-3-O-gallate (5.07 µg CA/g LP). Compound **19** was identified as (epi)catechin-3-*O*-coumarate, with a main fragment at 341 *m*/*z* obtained by heterolytic cleavage of the coumaric acid moiety [35]; however, this compound was present only in trace amounts. Procyanidins were identified based on characteristic fragments obtained by heterocyclic ring fission (loss of a phloroglucinol unit, −126 Da), retro-Diels–Alder fission (elimination of a hydroxyvinyl benzenediol unit, −152 Da); and quinone methide cleavage (cleavage of the interflavan bond) [64]. Procyanidin B-type dimers (**21**–**23**) and B-type trimers (**27**–**29**) were the most frequently detected, with the highest contents of procyanidin B2 (9.17 µg/g LP) and procyanidin C1 (8.11 µg/g LP), respectively. Compound **26** was identified as a procyanidin B-type gallate dimer (*m*/*z* 729) and was found at a content of 8.40 µg PB2/g LP. Other identified oligomeric derivatives included a chalcan-flavan-3-ol dimer (**24**) and an epicatechin ethyl dimer (**25**), but these compounds were present only in trace amounts (<LOQ). Detected flavonol aglycones (quercetin, kaempferol and isorhamnetin) were identified based on characteristic fragments obtained by *retro*-Diels–Alder cleavage and neutral losses (CH_3_, CO, CO_2_, H_2_O, C_2_H_2_O, and C_3_O_2_). Flavonol glycosides were mostly identified as monohexosides (**33–37**), with unique fragments (deprotonated aglycones) resulting by loss of a hexosyl unit (−162 Da). Compound **38** was identified as quercetin 3-*O*-(6″-*O*-rhamnosyl)hexoside, with main fragments at 300 *m*/*z* ([Y_0_–H]^–^) and 301 *m*/*z* ([Y_0_]^–^), obtained after loss of the rhamnosyl-hexosyl moiety (−308 Da). Quantification showed that all detected flavonols were present in trace amounts (<LOQ), except for quercetin-3-*O*-hexoside, which was found at a low content (0.51 µg KA/g LP). Resveratrol (**39**) and naringenin (**40**) were also identified and confirmed by comparison with available standards, but were present only in trace amounts. Finally, the total content of all detected phenolic compounds in the milk/ME/GPE powder was 215.32 µg/g LP.

In addition to phenolic compounds, phenylalanine and three hydroxylated fatty acids were detected as typical mushroom constituents, which previously were found and reported in *Agrocybe aegerita* [28], *Pleurotus ostreatus* [63] and/or *Tuber magnatum* [33]. Phenylalanine exhibited fragments at 147 *m*/*z* and 103 *m*/*z*, resulting from the sequential loss of NH_3_ (−17 Da) and CO_2_ (−44 Da). On the other hand, hydroxylated fatty acids showed characteristic fragments containing the carboxylate anion, generated *via* α-cleavage at the oxidized carbon [65]. In the absence of specific standards, these compounds were not quantified.

#### 3.3.7. Techno-Functional Properties of Optimized Milk/ME/GPE Powder

The results of EAI and ESI of 0.1% milk/ME/GPE solution are shown in Table 3. The milk/ME/GPE exhibited lower EAI and ESI compared to those previously reported for thermally treated skimmed goat milk [5], indicating a reduced emulsifying capacity. This behavior can be attributed to structural modifications of milk proteins induced by interactions with phenolic compounds and polysaccharides from the mushroom and grape pomace seed extracts. As confirmed by DLS analysis, the enriched system is characterized by increased particle size and a more complex, polymodal distribution, which reduces diffusion and adsorption efficiency of particles at the oil/water interface. Larger aggregates and protein–polyphenol complexes cover a smaller interfacial area, resulting in decreased EAI values, in agreement with previous findings [53]. Furthermore, electrophoretic analysis indicated partial proteolysis and the formation of lower-molecular-weight peptides, which may additionally influence protein conformation and interfacial behavior. These structural changes can alter the balance between hydrophilic and hydrophobic domains, reducing the ability of proteins to rapidly adsorb and stabilize newly formed interfaces. Polyphenols are known to bind proteins and can induce protein aggregation or reduced solubility, which may hinder their ability to adsorb at oil/water interfaces [57,66].

The ESI values, after initial reduction for around 44% after 10 min, remained unchanged after 30 min., suggesting the formation of a relatively stable interfacial film [5]. This may be explained by the formation of protein–polyphenol and protein–polysaccharide assemblies over the period of time, which can strengthen the interfacial film and contribute to the steric stabilization of oil droplets. Similar behavior has been reported for protein–phenolic systems, where conjugates improve emulsion stability despite reduced emulsifying activity [5].

Overall, the obtained results indicate that the addition of mushroom and grape pomace seed extracts leads to a complex modification of interfacial behavior: reducing emulsifying capacity (EAI) due to structural and size-related effects, while simultaneously contributing to emulsion stabilization through the formation of composite interfacial films.

In contrast to emulsifying properties, the milk/ME/GPE powder exhibited significantly higher FS and FC (Table 3) in comparison to those previously reported for thermally treated skimmed goat milk (2025b), indicating an improved ability to form and stabilize air bubbles. This behavior can be primarily attributed to structural modification of milk proteins induced by the presence of mushroom and grape pomace seed extracts. As shown by electrophoretic analysis, partial proteolysis of milk proteins resulted in the formation of lower-molecular-weight peptides. These smaller and more flexible protein structures are able to rapidly adsorb at the air/water interface, reducing surface tension more efficiently than intact casein micelles. In thermally treated skimmed goat milk, casein micelles are relatively large and structurally rigid, which limits their ability to rearrange and stabilize the air/water interface. Accordingly, poor foaming properties were previously observed for thermally treated skimmed goat milk [53]. Additionally, the presence of mushroom-derived polysaccharides, particularly β-glucans, likely contributes to foam stabilization by participating in the formation of a viscoelastic interfacial film around air bubbles. These components together with soluble protein fragments may enhance resistance to bubble coalescence and disproportionation, resulting in improved foam stability. The combined effect of flexible peptides and polysaccharide–protein interactions leads to the formation of a more cohesive and stable foam structure. It should be noted that phenolic compounds do not necessarily enhance foaming properties. Previous studies on goat milk systems enriched with grape pomace seed phenolics have reported reduced foaming capacity and stability, attributed to interactions between phenolics and proteins that can block active sites and decrease surface hydrophobicity [5]. Therefore, the improved foaming behavior observed in the present system is more plausibly related to protein hydrolysis and the presence of mushroom polysaccharides, rather than to protein–phenolic interactions alone. The mushroom proteins are known for good foaming properties. A recent study on ultrasound-extracted white button and oyster mushroom proteins reported remarkably high FC (82.5–235% across pH conditions) and substantial FS (up to 162.5%) [67].

The TM powder exhibited a higher WHC (1000 ± 0.1 g/100 g) compared to the milk/ME/GPE powder (899.5 ± 1.5 g/100 g). The reduction in WHC after enrichment can be related to structural modifications of milk proteins, as previously discussed. In particular, interactions with phenolic compounds and the formation of larger aggregates, confirmed by DLS analysis, may reduce the availability of hydrophilic sites and limit protein–water interactions, leading to lower WHC values. Previous studies have shown that the partial protein aggregation and binding of polyphenols to milk proteins can reduce the availability of hydrophilic sites and limit protein–water interactions, leading to lower WHC values [58,68,69].

A similar trend was observed for OHC, which was lower in the milk/ME/GPE powder (Table 3) compared to the TM (450 ± 1.5 g/100 g). This decrease may be associated with changes in protein conformation and surface properties, including reduced exposure of hydrophobic regions responsible for oil binding.

In contrast, previous studies on mushroom-fortified dairy systems have reported improved water retention, reduced syneresis, and higher WHC and OHC mainly attributed to the water-binding capacity of β-glucans and dietary fibers in mushrooms [3,70,71,72]. However, such effects appear to be system dependent. In the present study, the simultaneous presence of phenolic compounds, polysaccharides and structurally modified proteins likely resulted in the formation of a more compact or aggregated matrix, which limited both water and oil retention capacity.

#### 3.3.8. In Vitro Gastrointestinal Digestion of Functional Milk/ME/GPE Ingredient and Bioaccessibility of Phenolic Compounds

**Figure 5:**
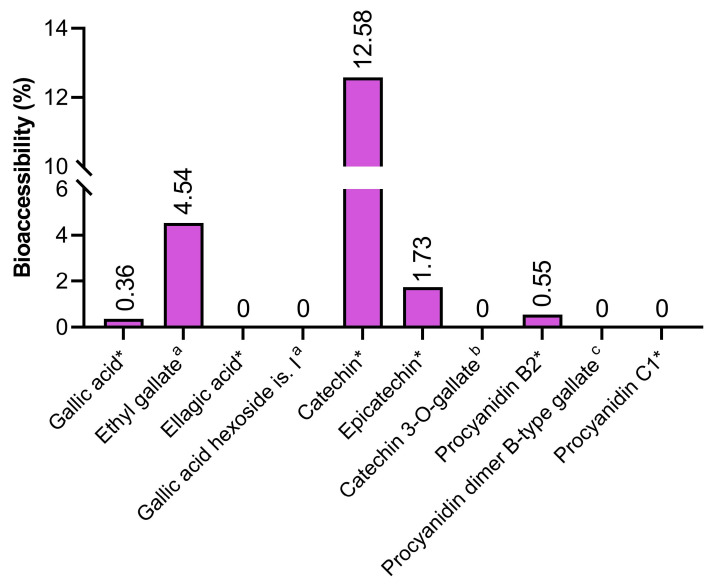
Bioaccessibility (%) of selected phenolic compounds after in vitro gastrointestinal digestion of milk/ME/GPE ingredient. Phenolic compounds were quantified/semi-quantified before and after in vitro gastrointestinal digestion, using UHPLC Q-ToF MS: * Compounds were quantified using reference standards; ^a^ Compound semi-quantified using gallic acid as standard; ^b^ Compound semi-quantified using catechin as standard; ^c^ Compound semi-quantified using procyanidin B2 as standard.

After in vitro gastrointestinal digestion (GID), selected phenolic compounds (Table 2) were monitored to assess their bioaccessibility and to evaluate the functional efficiency of the milk/ME/GPE ingredient. Among the analyzed compounds, catechin exhibited the highest bioaccessibility (12.58%), followed by ethyl gallate (4.54%) and epicatechin (1.73%) (Figure 5). Very low bioaccessibility was observed for gallic acid and procyanidin B2 (<1%), whereas the remaining compounds showed no detectable bioaccessibility because they were either present in trace amounts in the digesta (<LOQ or <LOD) or were not detected. Overall, all monitored phenolic compounds exhibited low bioaccessibility.

These findings are consistent with previous studies reporting low bioaccessibility of phenolic compounds, including catechin, epicatechin, procyanidins, gallic acid and its derivatives, following in vitro gastrointestinal digestion of grape seed extracts or powders and grape pomace extracts [29,73,74,75]. Previous studies have suggested that the bioaccessibility of phenolic compounds depends on several factors, including (a) the food matrix and its interactions with macronutrients such as proteins, carbohydrates, lipids and dietary fiber [76,77,78], (b) the release of phenolic compounds from the matrix and their solubilization during the intestinal phase [79], (c) interactions and complex formation with digestive enzymes and other constituents within the gastrointestinal tract [80], and (d) structural transformations induced by pH changes during digestion [81]. Complex food matrices, such as milk, bread, yogurt, egg-based products and infant formulas, have been shown to affect the release and solubility of phenolic compounds and to reduce the bioaccessibility of total phenolics, anthocyanins and/or procyanidins following in vitro gastrointestinal digestion [18,82,83]. The low bioaccessibility observed in the present study may be related to the presence of milk proteins, which have a strong tendency to interact with phenolic compounds [18].

Consequently, the phenolic compounds may remain associated with the insoluble fraction and could potentially become available during further gastrointestinal processing and microbial metabolism in the colon [77]. Sun and Cheng [84] demonstrated the high bioaccessibility of phenolic compounds from wine after the colon phase of gastrointestinal digestion and strong biological activity.

### 3.4. Physicochemical Characterization of Fortified Soup

The physicochemical properties of the control (C) and fortified (S) soup powders are presented in Table 4. The incorporation of the milk/ME/GPE functional ingredient resulted in only minor modifications of the overall powder characteristics.

As shown in Table 4, the fortified soup powder (S) exhibited a slightly lower moisture content compared to the control soup powder (C), indicating that the addition of extracts reduced retained water. In similar works, spray-dried grape skin-whey protein powders and spray-dried mushroom-whey soup powder yielded low moisture values [85,86], which is essential for ensuring microbiological stability and extended shelf life [87,88].

Water solubility values were very similar for both the S and C samples, indicating that the addition of the functional ingredient did not adversely affect the dissolution behavior of the powder. This suggests that the dominant matrix components, such as milk proteins and starch, govern the solubility of the system, while the incorporated extracts do not significantly interfere with this property. Similar observations have been reported for multicomponent food powders, where the presence of bioactive compounds does not necessarily alter bulk solubility but may influence other aspects of rehydration [87]. The water solubility of around 47% is due to the solid soup ingredients such as onion, garlic and black pepper that disperse into the water. Wettability was essentially unchanged, indicating that surface wetting of S was not markedly different from C.

For both soups, the formation of lumps on the surface of the water was observed which can be attributed to the property of water to poorly wet very fine powders due to high surface tension [87]. Furthermore, the addition of the functional ingredient did not significantly modify surface properties such as hydrophilicity or particle porosity, which are known to influence the wetting process. According to the literature, wettability is mainly governed by the surface properties of powder particles, whereas structural features such as porosity and particle size can additionally influence rehydration behavior for milk-based powders [87]. The absence of differences between the samples indicates that these parameters remained within a similar range despite formulation changes. Similar results for wettability of soymilk-based powder were shown in the study by Jinapong et al. [89].

The bulk and tapped density influences the required storage volume and plays a key role in determination of the packaging expenses and transport of the dried products [90]. These values did not differ significantly among the tested samples. The values of bulk density of most food powders are within the range of 0.3–0.8 g/cm^3^ [90]. Based on the compressibility index (CI), it was determined that the flowability of the tasted powders was fair, while the cohesiveness of the control soup (C) was intermediate, and that of the functional soup (S) was high [89,91]. Fair flowability can be attributed to small particle size that has large surface per unit mass of powder enabling cohesive forces that resist flow. Addition of the functional ingredient changed particle sizes and particle surface properties leading to an increase in cohesive forces. Similar observation was reported by Jinapong, Suphantharika and Jamnong [89].

### 3.5. Sensory and Antioxidant Properties of Fortified Soups

A prepared soup with an optimized mix of added dehydrated vegetables and a functional ingredient was compared to a commercial soup that has been on the market for many years. The commercial soup, besides dehydrated vegetables, contains flavor enhancers (monosodium glutamate, MSG), added flavors, dextrose for a hint of sweetness, milk protein hydrolysates for flavor body and a range of spices (parsley, oregano, bay leaf, pepper, chives, basil, rosemary, etc.) as listed in the Material and Methods.

#### 3.5.1. Overall Quality Evaluation

**Figure 6:**
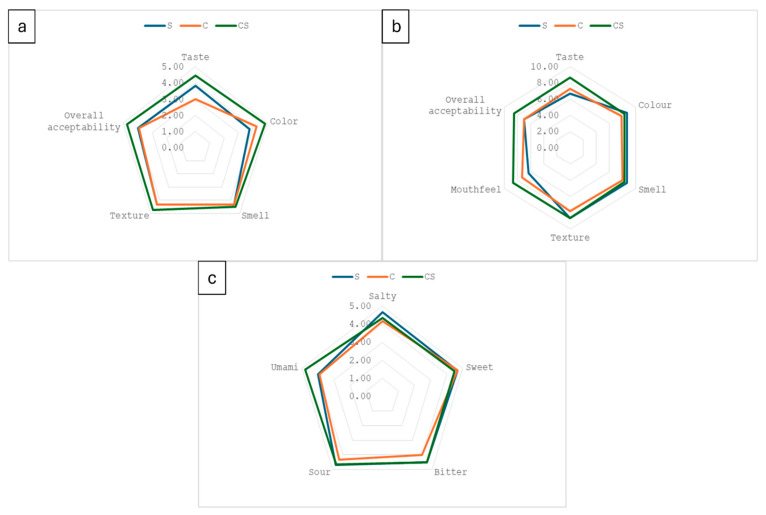
(**a**) Sensory analysis of formulated dehydrated soups obtained by overall quality evaluation. (**b**) Sensory analysis of formulated dehydrated soups obtained by overall quality evaluation for taste quality parameters. (**c**) Sensory analysis of formulated dehydrated soups obtained by consumer acceptance evaluation. Abbreviations: S (fortified soup)—12.25% garlic powder, 12.25% onion powder, 10.5% black pepper, 11.9% sea salt, 30% starch, and 17.5% functional ingredient with milk/ME/GPE; C (control soup)—12.25% garlic powder, 12.25% onion powder, 10.5% black pepper, 11.9% sea salt, 35.6% starch, and 17.5% thermally treated defatted goat milk; CS—Quik e-free instant fat-free vegetable soup, Aleva, Serbia.

The results of sensory analysis obtained by overall quality evaluation are presented in Figure 6a. As expected, the commercial soup received the highest scores for color, smell, taste, texture and overall acceptability (Figure 6a). The formulated soup with added mushroom and grape pomace seed extracts achieved an overall acceptability score of 4.00 out of 5.00, which is a promising result for a new formulation.

When comparing the taste attribute ratings (salty, sweet, bitter, sour, umami) of the soup samples (Figure 6b), all samples had similar scores for these four taste characteristics. The only exception was the umami taste. Here the commercial sample (CS) scored the highest (4.80) compared to 4.00 for sample S. This indicates that the commercial soup delivered a stronger umami sensation. Mushrooms are known to contribute significantly to umami taste in plant-based foods due to their high content of free amino acids (e.g., glutamic acid) and other compounds (e.g., ribonucleotides), which impart a savory, meat-like flavor [92]. In line with this, sample S exhibited a more pronounced umami flavor than the control sample (C). In fact, mushroom extracts have been investigated as natural flavor enhancers and can even serve as alternatives to monosodium glutamate (MSG) due to their rich umami taste and aroma profile [93]. The term umami denotes a pleasant savory taste, unlike other basic tastes, umami is sensed across the entire tongue and is key to the palatable richness of many foods.

#### 3.5.2. Consumer Acceptance Evaluation

Using a nine-point hedonic scale (Figure 6c), the formulated sample S was rated highest for color and smell (8.67), and its texture received a relatively high score and was well accepted by consumers. For mouthfeel, however, the commercial sample scored the highest (8.66), followed by the sample containing only dehydrated goat milk (7.33), with sample S scoring lower in comparison. The overall acceptability was greatest for sample CS, as expected, while the formulated samples S and C both scored 7.00 for overall acceptability.

Based on the results, it can be concluded that each added ingredient contributed to specific aspects of the soup’s sensory properties. The addition of grape pomace seed extract in the formulation S improved the soup texture, consistent with studies reporting that grape seed polyphenols can enhance or preserve textural quality in fortified foods [94]. The inclusion of mushroom extract in sample S intensified the aroma profile of the soup, as mushrooms contain distinctive volatile compounds (ketones, esters) that boost savory aromas [93,95]. Additionally, the incorporation of dehydrated goat milk contributed to the taste and mouthfeel. Goat milk is rich in short- and medium-chain fatty acids that impart a distinctive, full-bodied flavor complexity [96], thereby enriching the overall taste of sample C.

Thanks to these contributions, the formulated samples achieved relatively high average scores on sensory evaluation. Notably, both sensory evaluation methods revealed the same trend in preferences. The commercial soup (CS) received the best score, which was anticipated given its flavor-enhancing ingredients. The consumer acceptance and overall quality testing gave very similar outcomes for all samples, indicating reliable and colaborating sensory results across methods. Importantly, the novel formulated soup with milk/ME/GPE was sensorially acceptable under the tested conditions, particularly for umami taste, smell and texture without any added fats, artificial flavorings or flavor enhancers. The formulated soup may serve as a basis for the development of an innovative product while maintaining acceptable sensory properties.

#### 3.5.3. Antioxidant Properties of Digested Fortified Soup

**Figure 7:**
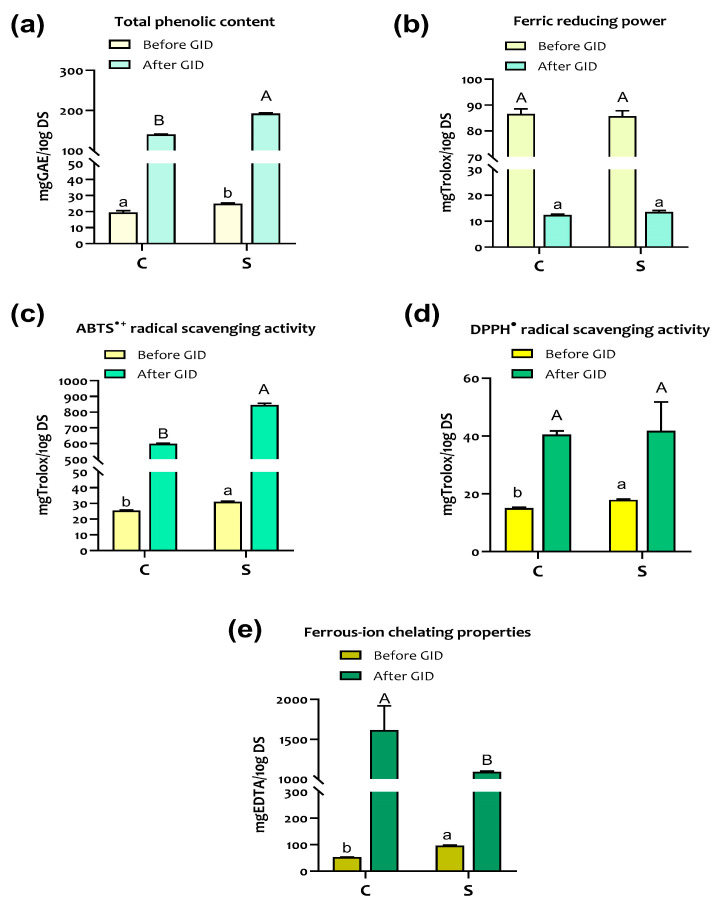
Antioxidant properties of control and fortified soups before and after in vitro gastrointestinal digestion: (**a**) total phenolic content; (**b**) ferric reducing power; (**c**) ABTS^•+^ radical scavenging activity; (**d**) DPPH^•^ radical scavenging activity; (**e**) ferrous chelating properties. Abbreviations: C—control soup; S—fortified soup. The comparison between means before digestion is indicated by small case letters. The comparison between means after digestion is indicated by upper case letters. The means with the same letters are not statistically different ar *p* < 0.05.

To provide additional evidence supporting the functional potential of the developed functional ingredient, antioxidant properties of control and fortified soup before and after in vitro gastrointestinal digestion were evaluated. The obtained results are presented in Figure 7.

Prior to digestion, the fortified soup exhibited higher radical scavenging activity and ferrous-ion chelating capacity than the control soup. Following in vitro gastrointestinal digestion, both soups showed a significant increase in antioxidant properties measured by TPC, ABTS, DPPH and CHE tests, except FRP compared with the corresponding undigested samples, indicating the release and/or improved accessibility of antioxidant compounds during digestion. Considering that the control soup did not contain the functional ingredient, the increased antioxidant properties can be attributed to the antioxidant constituents originating from garlic, onion and black pepper present in the soup formulation.

After digestion, the enriched soup exhibited significantly higher total phenolic content (TPC) and ABTS radical-scavenging activity than the control soup. In contrast, no significant differences were observed between the soups in DPPH radical-scavenging activity, ferric reducing power (FRP), or ferrous-ion chelating ability (CHE). The increased antioxidant properties observed after digestion may be related to the release of grape phenolic compounds (Figure 5), bioactive peptides liberated during protein digestion, mushroom-derived β-glucans, as well as antioxidant constituents originating from garlic, onion and black pepper present in the soup formulation. In addition, gastrointestinal digestion may increase the accessibility of matrix-bound bioactive compounds. Consequently, the measured antioxidant activity may represent the combined effect of multiple classes of compounds released or becoming more accessible during digestion.

Similar observations have been reported for food matrices enriched with grape-derived phenolic compounds, where gastrointestinal digestion resulted in increased antioxidant activity despite substantial changes in the profile and bioaccessibility of individual phenolic compounds. Such effects have been attributed to the combined contribution of released phenolic compounds and digestion-derived hydrolysates originating from the food matrix [83].

### 3.6. Perspectives for Industrial Application

The developed ingredient is composed of components originating from edible sources with a documented history of food use, including goat milk, *Agrocybe aegerita* mushroom and grape pomace seed extracts. From a regulatory perspective, the commercialization of such multifunctional ingredients requires consideration of ingredient standardization, compositional consistency, safety assessment and compliance with the applicable food legislation. Although both grape-derived ingredients and edible mushrooms have been extensively used in food systems, the final regulatory classification of concentrated extracts may depend on their composition, manufacturing process and intended use.

In addition, the safety and quality of the developed dehydrated soup system should be further evaluated through storage stability studies, microbiological assessment and shelf-life determination. Future investigations should also address the stability and bioaccessibility of bioactive compounds during storage and digestion in order to support potential health-related claims and industrial implementation. Nevertheless, the results obtained in the present study provide a scientific basis for the development of novel milk-based functional ingredients enriched with natural bioactive compounds from grape pomace and edible mushrooms.

## 4. Conclusions

This study demonstrated the successful development of a novel goat milk-based functional ingredient enriched with *Agrocybe aegerita* mushroom extract and Prokupac grape pomace seed extract. The application of a central composite design enabled preliminary optimization of the formulation and provided insight into the influence of formulation variables on the antioxidant properties of the developed ingredient. Among the investigated factors, grape pomace seed extract was identified as the dominant factor influencing antioxidant properties.

Although moderate deviations between predicted and experimental values were observed, particularly for ABTS*+ and DPPH* assays, the developed models provided a useful tool for preliminary formulation optimization and evaluation of general response trends, highlighting the complexity of antioxidant evaluation in multicomponent food systems.

The combined application of ATR-FTIR, DLS and SDS-PAGE provided complementary insights into the structural organization of the system. While ATR-FTIR spectra were dominated by milk constituents and did not reveal distinct new bands, DLS and electrophoretic analysis revealed structural modifications, including polymodal particle size distribution and alterations in the protein profile. These observations may be associated with protein–polyphenol interactions, structural rearrangements within the matrix and/or partial protein hydrolysis. These results emphasize that the absence of clear spectral changes in ATR-FTIR does not necessarily exclude the occurrence of molecular interactions, but rather reflects the limitations of single analytical techniques when applied to complex food matrices.

The incorporation of mushroom and grape pomace seed extracts significantly modified the techno-functional properties of the goat milk matrix. Enhanced antioxidant properties and improved foaming behavior were accompanied by a reduction in emulsifying capacity, reflecting a technological trade-off that may be associated with protein structural modification of milk proteins and intermolecular interactions within the system. Therefore, the suitability of the developed ingredient may depend on the requirements of the intended food application.

Furthermore, the developed ingredient demonstrated good applicability in a dehydrated soup model, maintaining satisfactory solubility, physicochemical stability, rehydration behavior and sensory acceptability under the conditions tested. The enriched soup also displayed enhanced antioxidant properties after simulated gastrointestinal digestion, further supporting its potential as a functional food ingredient.

Overall, this study highlights the potential of combining dairy proteins with plant- and mushroom-derived bioactives for the development of novel food ingredients with enhanced antioxidant potential. The results also underscore the importance of integrated analytical approaches for comprehensive understanding of structural and functional changes occurring in complex food systems. Future studies should evaluate storage stability, microbiological safety and shelf-life characteristics of the developed dehydrated soup system.

## Figures and Tables

**Table 1:** Predicted and experimental results of analyzed responses *.

Response	Individual Desirability	Predicted Value	Obtained Value	Recovery, %
TPC (mg GAE/100 mL)	0.852107	95.15	118.6	125
ABTS*+ (mg Trolox/100 mL)	0.967743	108.0	62.78	58
DPPH* (mg Trolox/100 mL)	1.000000	59.90	129.0	215

* Total phenolic content—TPC; ABTS*+ scavenging activity—ABTS*+; DPPH* scavenging activity—DPPH*.

**Table 2:** Untargeted UHPLC Q-ToF MS profiling and quantification/semi-quantification of phenolic compounds in methanolic extract of optimized milk/ME/GPE powder (0.5% GPE, 0.5% ME, pH = 6.5). Target compounds, expected retention time (RT), molecular formula, calculated mass, *m*/*z* exact mass and MS fragments are presented in the table.

No.	RT	Compound Name	Formula	Calculated Mass	*m*/*z* Exact Mass	mDa	Fragments (MS^2^)	µg/g LP
Phenolic acids and derivatives ^a^								
1	4.52	Hydroxybenzoic acid	C_7_H_5_O_3_^—^	137.0239	137.0240	−0.09	/	3.95
2	6.25	Dihydroxybenzoic acid	C_7_H_5_O_4_^—^	153.0188	153.0188	−0.05	**109.0336(100)**, 108.0244,	19.24
3	1.34	Gallic acid *	C_7_H_5_O_5_^—^	169.0137	169.0145	−0.81	**125.0278(100)**, 124.0202	47.78
4	5.86	Methyl gallate	C_8_H_7_O_5_^—^	183.0293	183.0284	0.93	**124.0206(100)**, 125.0243	<LOQ
5	7.42	Ethyl gallate	C_9_H_9_O_5_^—^	197.0450	197.0467	−1.72	**124.0205(100)**, 125.0272, 169.0189	54.05
6	7.88	Ellagic acid *	C_14_H_5_O_8_^—^	300.9984	301.0042	−5.80	**301.0071(100)**, 299.9992, 145.0336, 157.0346, 185.0292, 201.0250, 229.0202, 245.0152, 257.0160	11.70
7	4.47	Vanilloloside	C_14_H_19_O_8_^—^	315.1080	315.1104	−2.39	**123.0493(100)**, 153.0603	<LOQ
8	3.36	Galloylshikimic acid	C_14_H_13_O_9_^—^	325.0560	325.0598	−3.79	**125.0273(100)**, 111.0504, 124.0195, 137.0245, 168.0067, 169.0184	<LOQ
9	2.09	Gallic acid hexoside is. I	C_13_H_15_O_10_^—^	331.0665	331.0712	−4.73	**169.0201(100)**, 168.0112, 125.0276, 124.0209	5.58
10	3.13	Gallic acid hexoside is. II	C_13_H_15_O_10_^—^	331.0665	331.0728	−6.32	**169.0194(100)**, 124.0187, 125.0287	7.58
11	5.99	Syringic acid hexoside	C_15_H_19_O_10_^—^	359.0978	359.1079	−10.07	**197.0514(100)**, 137.0280, 138.0362, 239.0607	<LOD
12	7.61	Ellagic acid pentoside	C_19_H_13_O_12_^—^	433.0407	433.0490	−8.33	**299.9967(100)**, 301.0054	3.43
13	5.52	Digalloyl hexoside is. I	C_20_H_19_O_14_^—^	483.0775	483.0861	−8.64	**169.0174(100)**, 124.0177, 125.0292, 313.0637, 331.0721	<LOD
14	5.87	Digalloyl hexoside is. II	C_20_H_19_O_14_^—^	483.0775	483.0863	−8.79	**169.0185(100)**, 125.0269, 313.0632, 331.0730	<LOQ
15	8.34	Dicaffeoylquinic acid	C_25_H_23_O_12_^—^	515.1190	515.1286	−9.63	**191.0624(100)**, 135.0501, 161.0304, 173.0525, 179.0409, 353.0978	<LOD
16	6.54	Galloyl-HHDP-hexose	C_27_H_21_O_18_^—^	633.0728	633.0862	−13.42	**301.0058(100)**, 169.0185, 463.0619	<LOD
		∑						153.32
Flavan-3-ols and derivatives ^b^								
17	6.33	Catechin *	C_15_H_13_O_6_^—^	289.0712	289.0756	−4.40	**123.0493(100)**, 109.0334, 125.0286, 137.0289, 151.0447, 161.0646, 187.0459, 203.0771, 221.0879	13.36
18	7.00	Epi-catechin *	C_15_H_13_O_6_^—^	289.0712	289.0751	−3.90	**109.0336(100)**, 123.0494, 125.0289, 137.0291, 149.0298, 151.0452, 161.0655, 203.0776, 221.0886	5.08
19	8.89	(Epi)catechin-3-*O*-coumarate	C_24_H_19_O_8_^—^	435.1080	435.1189	−10.89	**341.0761(100)**, 109.0326, 123.0487, 145.0339, 189.0245, 217.0207	<LOD
20	7.95	Catechin-3-*O*-gallate	C_22_H_17_O_10_^—^	441.0822	441.0908	−8.59	**169.0194(100)**, 109.0332, 125.0286, 137.0288, 203.0771, 245.0889, 289.0801	5.07
		∑						23.50
Procyanidins ^c^								
21	6.13	Procyanidin dimer B type is. I	C_30_H_25_O_12_^—^	577.1346	577.1447	−10.07	**289.0799(100)**, 125.0285, 137.0287, 161.0296, 203.0764, 245.0880, 339.0963, 407.0880	5.74
22	6.80	Procyanidin B2 *	C_30_H_25_O_12_^—^	577.1346	577.1454	−10.80	**289.0797(100)**, 109.0330, 123.0492, 125.0284, 137.0288, 203.0770, 245.0888, 407.0870	9.17
23	7.34	Procyanidin dimer B type is. II	C_30_H_25_O_12_^—^	577.1346	577.1464	−11.84	**289.0798(100)**, 125.0285, 137.0287, 161.0294, 245.0875, 273.0483, 290.08286(19), 299.06485(6), 407.08847(49)	1.96
24	7.01	Chalcan-flavan-3-ol dimer	C_30_H_27_O_12_^—^	579.1503	579.1586	−8.25	**289.0799(100)**, 109.0333, 123.0494, 125.0285, 137.0289, 161.0643, 203.0773, 245.0893	<LOD
25	9.03	Ethyl (epi)catechin-(epi)catechin (Epicatechin ethyl dimer)	C_32_H_29_O_12_^—^	605.1659	605.1738	−7.87	**289.0798(100)**, 109.0312, 125.0279, 137.0289, 205.0567, 229.0937, 245.0895, 315.0967	<LOD
26	7.41	Procyanidin dimer B type gallate	C_37_H_29_O_16_^—^	729.1456	729.1588	−13.19	**407.0886(100)**, 125.0284, 169.0195, 289.0799, 408.0914, 425.0988, 441.0932, 451.1151, 577.1446	8.40
27	6.60	Procyanidin trimer B type is. I	C_45_H_37_O_18_^—^	865.1980	865.2159	−17.91	**287.0642(100)**, 289.0794, 125.0283, 161.0291, 407.0884, 425.0988, 451.1144, 575.1338, 577.1495, 695.1576, 713.1683	2.62
28	7.27	Procyanidin C1 *	C_45_H_37_O_18_^—^	865.1980	865.2139	−15.88	**287.0641(100)**, 289.0792, 125.0283, 161.0294, 407.0879, 425.0991, 449.0992, 451.1154, 575.1335, 577.1495, 695.1574, 713.1683	8.11
29	7.81	Procyanidin trimer B type is. II	C_45_H_37_O_18_^—^	865.1980	865.2136	−15.62	**287.0632(100)**, 289.0791, 125.0276, 407.0867, 425.0966, 449.0968, 451.1139, 575.1324, 577.1465, 695.1555	2.01
		∑						37.99
Flavanol aglycones and glycosides ^d^								
30	10.37	Kaempferol *	C_15_H_9_O_6_^—^	285.0399	285.0444	−4.48	**285.0485(100)**, 107.0174, 143.0548, 145.0342, 151.0084, 157.0694, 185.0662, 211.0469, 227.0419, 229.0573, 239.0414	<LOD
31	9.57	Quercetin *	C_15_H_9_O_7_^—^	301.0348	301.0399	−5.07	**151.0085(100)**, 107.0177, 121.0334, 179.0039, 245.0528, 273.0484	<LOD
32	10.51	Isorhamnetin	C_16_H_11_O_7_^—^	315.0505	315.0554	−4.93	**300.0366(100)**, 301.0383, 107.0173, 151.0084, 164.0174, 216.0495, 227.0428, 255.0354, 271.0326	<LOD
33	8.29	Kaempferol 3-*O*-hexoside	C_21_H_19_O_11_^—^	447.0927	447.1012	−8.52	**284.0408(100)**, 285.0467, 151.0084, 227.0415, 255.0374	<LOD
34	7.95	Quercetin-3-*O*-hexoside	C_21_H_19_O_12_^—^	463.0877	463.0973	−9.58	**300.0367(100)**, 301.0433, 151.0085, 179.0045, 243.0378, 271.0331	0.51
35	8.42	Isorhamnetin-3-*O*-hexoside	C_22_H_21_O_12_^—^	477.1033	477.1071	−3.78	**314.0524(100)**, 315.0571, 151.0085, 271.0327, 300.0352	<LOQ
36	7.48	Myricetin 3-*O*-hexoside	C_21_H_19_O_13_^—^	479.0826	479.0957	−13.15	**316.0312(100)**, 317.0362, 151.0082, 179.0039, 271.0318	<LOD
37	8.42	Syringetin 3-O-hexoside	C_23_H_23_O_13_^—^	507.1139	507.1247	−10.83	**344.0615(100)**, 329.0270, 330.0493, 316.0636, 301.0685, 273.0434	<LOQ
38	7.81	Quercetin 3-*O*-(6”-*O*-rhamnosyl) hexoside	C_27_H_29_O_16_^—^	609.1457	609.1504	−4.76	**300.0358(100)**, 301.0425, 151.0082, 179.0039, 255.0369, 271.0315, 609.1611	<LOD
Other phenolic compounds								
39	8.96	Resveratrol *	C_14_H_11_O_3_^—^	227.0708	227.0756	−4.83	**143.0542(100)**, 117.0386, 157.0706, 159.0866, 183.0851, 185.0672	<LOD
40	10.17	Naringenin *	C_15_H_11_O_5_^—^	271.0606	271.0624	−1.85	**119.0548(100)**, 107.0127, 151.0086	<LOD
		∑∑ phenolic compounds						215.32
Amino acids and Fatty acids								
41	1.78	Phenylalanine	C_9_H_10_NO_2_^—^	164.0712	164.0772	−6.05	**103.0589(100)**, 147.0499	nq
42	14.82	Hydroxyoctadecadienoic acid	C_18_H_31_O_3_^—^	295.2273	295.2355	−8.18	**183.1076(100)**, 139.1146, 155.1132, 171.1080, 195.1446, 277.2251	nq
43	13.20	Dihydroxyoctadecenoic acid	C_18_H_33_O_4_^—^	313.2379	313.2401	−2.22	**183.1432(100)**, 129.0948, 195.143, 297.1936	nq
44	10.77	Trihydroxyoctadecenoic acid	C_18_H_33_O_5_^—^	329.2328	329.2424	−9.60	**171.1078(100)**, 127.1162, 139.1171, 155.1119, 183.1461, 201.1181, 211.1380, 229.1521, 311.2397, 293.2218	nq

**Abbreviations:** GPE—Prokupac grape pomace seed extract; ME—*Agrocybe aegerita* mushroom extract; LP—lyophilized powder; nq—not quantified compounds (content of these compounds was not determined); <LOD—less of limit of detection; <LOQ—less of limit of quantification. (*) Compounds were quantified using reference standards; (^a^) compound content expressed in gallic acid equivalents (µg GA/g LP); (^b^) compound content expressed in catechin equivalents (µg CA/g LP); (^c^) compound content expressed in procyanidin B2 equivalents (µg PB2/g LP); (^d^) compound content expressed in kaempferol equivalents (µg KA/g LP): nq not quantified.

**Table 3:** Techno-functional properties of milk/ME/GPE ingredient. Abbreviations: emulsion activity index (EAI); emulsion stability index (ESI) after 10 and 30 min; foam stability (FS); foam capacity (FC); water holding capacity (WHC); oil holding capacity (OHC).

Sample	EAI (%)	ESI (%)		FC (%)	FS (%)	WHC (g/100 g)	OHC (g/100 g)
		10 min	30 min				
milk/GPE/ME	83.3 ± 0.8	56.3 ± 2.95 ^a^	52.1 ± 2.95 ^a^	225 ± 5	75 ± 3.5	899.5 ± 1.5	386.08 ± 2.5

milk/ME/GPE—thermally treated milk/mushroom extract/grape pomace seed extract. The comparison between means is indicated by case letters. The means with the same letters are not statistically different at *p* < 0.05.

**Table 4:** The moisture content, water solubility, wetting rate, bulk density, tapped density, compressibility and Hausner ratio of functional soup.

Samples	Moisture (%)	Water Solubility (%)	Wetting Rate (s)	The Bulk Density (g/mL)	The Tapped Density (g/mL)	The Compressibility Index (CI, %)	Hausner Ratio (HR)
S	14.02 ± 0.11 ^a^	46.5 ± 0.05 ^a^	57 ± 3 ^a^	0.40 ± 0.00 ^a^	0.57 ± 0.00 ^a^	30 ± 1 ^a^	1.43 ± 0.01 ^a^
C	13.34 ± 0.13 ^b^	47.1 ± 0.04 ^b^	57 ± 3 ^a^	0.45 ± 0.00 ^b^	0.59 ± 0.00 ^b^	23 ± 0 ^b^	1.29 ± 0.00 ^b^

Abbreviations: C (control soup)—12.25% garlic powder, 12.25% onion powder, 10.5% black pepper, 11.9% sea salt, 35.6% starch, and 17.5% thermally treated defatted goat milk; S (fortified soup)—12.25% garlic powder, 12.25% onion powder, 10.5% black pepper, 11.9% sea salt, 30% starch, and 17.5% functional ingredient with milk/ME/GPE. The comparison between means is indicated by case letters. The means with the same letters are not statistically different at *p* < 0.05.

## Data Availability

The original contributions presented in this study are included in the article/Supplementary Materials. Further inquiries can be directed to the corresponding authors.

## Supplementary Materials

The following supporting information can be downloaded at https://www.mdpi.com/article/10.3390/foods15132397/s1, Table S1: Experimental factors and levels of factors; Table S2: Central Composite Design and real values; Table S3: Regression equation and other accompanying parameters for standards used for quantification or semi-quantification of (tentatively) identified phenolic compounds in methanolic extract of optimized milk/ME/GPE powder; Table S4: Estimated regression coefficients and analysis of variance for total phenolic content (Folin–Ciocalteu method—TPC) response design; Table S5: Estimated regression coefficients and analysis of variance for ABTS*+ (ABTS*+ radical scavenging activity) response design; Table S6: Estimated regression coefficients and analysis of variance for DPPH* (DPPH* radical scavenging activity) response design; Table S7: Estimated regression coefficients and analysis of variance for ferric reducing power (FRP) response design; Table S8: Estimated regression coefficients and analysis of variance for ferrous chelating capacity (CHE) response design; Table S9: Second-order polynomial equations and regression coefficient of the response variables*; Table S10: Dataset for overall optimization*. Table S11. The polypeptide composition of the optimized milk/ME/GPE and control TM powders, analyzed by SDS-PAGE in reducing conditions (SDS-R-PAGE). Figure S1. Electrophoregrams of polypeptide profile of the optimized milk/ME/GPE and control TM powders, analyzed by SDS-PAGE in reducing conditions (SDS-R-PAGE).
