# Supplementary material for: Development of Skimmed Goat Milk Functional Ingredient Enriched with Grape Pomace Seed and Agrocybe aegerita Extracts: Optimization, Characterization and Application in Dehydrated Foods
Source: Foods. 2026 Jul 6;15(13):2397. doi: 10.3390/foods15132397 (PMC13361018; doi:10.3390/foods15132397)
Supplement: Supplementary file 1 [file foods-15-02397-s001.zip › foods-4372679-supplementary.pdf]

Supplementary material

Development of Skimmed Goat Milk Functional Ingredient Enriched with Grape Pomace Seed and *Agrocybe aegerita* Extracts: Optimization, Characterization and Application in Dehydrated Foods

Ana Plećić [1,†], Danijel D. Milinčić [1,†], Ivana Sredović Ignjatović [1], Jovana Petrović [2], Aleksandar Ž. Kostić [1], Ana Doroški Petković [1], Steva M. Lević [1], Slađana P. Stanojević [1], Vladimir B. Pavlović [1], Vladislav Rac [1], Viktor A. Nedović [1,*] and Mirjana B. Pešić [1,*]

[1] Department of Food Technology and Biochemistry, Faculty of Agriculture, University of Belgrade,
Nemanjina 6, 11080 Belgrade, Serbia; ana.bjekovic@agrif.bg.ac.rs (A.P.); danijel.milincic@agrif.bg.ac.rs (D.D.M.); isredovic@agrif.bg.ac.rs (I.S.I.); akostic@agrif.bg.ac.rs (A.Ž.K.); ana.doroski@agrif.bg.ac.rs (A.D.P.); slevic@agrif.bg.ac.rs (S.M.L.); sladjas@agrif.bg.ac.rs (S.P.S.); vlaver@agrif.bg.ac.rs (V.B.P.); vladarac@agrif.bg.ac.rs (V.R.)

[2] Department of Plant Physiology, National Institute of Republic of Serbia, Institute for Biological Research "Siniša Stanković", University of Belgrade, 11060 Belgrade, Serbia; jovana0303@ibiss.bg.ac.rs

* Correspondence: vnedovic@agrif.bg.ac.rs (V.A.N.); mpesic@agrif.bg.ac.rs (M.B.P.); Tel.: +381-114413315 (V.A.N.); 381-114413468 (M.B.P.)

† These authors contributed equally to this work.

Table S1. Experimental factors and levels of factors.

Factor	-1 (Low)	0 (Medium)	+1 (High)
w (ME), % (m/m)	0.1	0.3	0.5
w (GPE), % (m/m)	0.1	0.3	0.5

Table S2. Central Composite Design and real values.

Exp.	*w*(ME), %	*w*(GPE), %
1	0.3	0.1
2	0.1	0.1
3	0.5	0.1
4	0.1	0.3
5	0.5	0.5
6	0.3	0.3
7	0.3	0.3
8	0.1	0.5
9	0.3	0.5
10	0.3	0.3
11	0.3	0.3
12	0.5	0.3
13	0.3	0.1

Table S3. Regression equation and other accompanying parameters for standards used for quantification or semi-quantification of (tentatively) identified phenolic compounds in methanolic extract of optimised milk/ME/GPE powder.

Standards	Regression equation Y = a*X ± b	R^2	*r*	Linear range (ppm)	LOD (ppm)	LOQ (ppm)
	Phenolic compound standards					
Gallic acid	y = 28494044.8850x + 114785.5339	0.9996	0.9998	0.0039-0.25	0.006	0.019
Resveratrol	y = 24658918.0674x + 118898.1926	0.9992	0.9996	0.0039-0.125	0.005	0.015
Naringenin	y = 311153373.4202x + 499627.5632	0.9987	0.9993	0.0039-0.0625	0.003	0.010
Kaempferol	y = 294203327.5794x + 156236.8726	0.9998	0.9999	0.0039-0.0625	0.001	0.004
Catechin	y = 43043679.1425x + 143310.4885	1.0000	1.0000	0.0039-0.125	0.001	0.003
Epicatechin	Y = 70882446.3869 * X + 263231.7974	0.9996	0.9998	0.0039-0.0625	0.002	0.005
Ellagic acid	Y = 73889226.5536 * X + 386690.6613	0.9997	0.9998	0.0039-0.25	0.006	0.017
Quercetin	Y = 290676924.1270 * X + 498112.4361	0.9991	0.9995	0.0039-0.0625	0.003	0.008
Procyanidin B2	Y = 34320698.5984 * X + 145091.6422	1.0000	1.0000	0.0039-0.25	0.002	0.006
Procyanidin C1	Y = 15688495.6998 * X + 152899.8761	0.9986	0.9993	0.0039-0.25	0.012	0.037

Abbreviations: R^2-coefficient of determination; *r*-correlation coefficient; LOD-limit of detection (3:1 Signal-to-noise ratio); LOQ-limit of quantification (10:1 Signal-to-noise ratio); %RSD-percent relative standard deviation. **Equations:**

$$r = \sqrt{R^1} \qquad (a);$$

$$LOQ = \frac{3.3xSEa}{S} \qquad (b);$$

$$LOQ = \frac{10xSEa}{S} \qquad (c);$$

Where; *r*- correlation coefficient; R^2-coefficient of determination; LOD-limit of detection; SEa-standard error of the y-intercept of the calibration curve; S- slope of the calibration curve.

Table S4. Estimated regression coefficients and analysis of variance for total phenolic content (Folin Ciocalteu method – TPC) response design.

Estimated regression coefficients for the response surface design

Term	Coefficient	Standard error of the coefficient	*p*-value
Constant	57.1845	5.496	0.000
w(ME), %	0.7733	5.403	0.890
w(GPE), %	19.4883	5.403	0.009
w(ME) * *w*(ME)	2.1293	7.964	0.797
w(GPE) * *w*(GPE)	11.5143	7.964	0.191
w(ME) * *w*(GPE)	4.0650	6.618	0.558

Analysis of variance (ANOVA) for response surface model (coded units)

Source of variation	Degrees of freedom	Sequential sum of squares	Adjusted sum of squares	Adjusted mean of squares	*F*-value	*p*-value
Regression	5	2851.79	2851.79	570.36	3.26	0.078
Linear	2	2282.36	2282.36	1141.18	6.51	0.025
Square	2	503.33	503.33	251.67	1.44	0.300
Interaction	1	66.10	66.10	66.10	0.38	0.558
Residual Error	7	1226.23	1226.23	175.18		
Lack-of-Fit	3	265.58	265.58	88.53	0.37	0.781
Pure Error	4	960.64	960.64	240.16		
Total	12	4078.02				

Table S5. Estimated regression coefficients and analysis of variance for ABTS*+ (ABTS*+ radical scavenging activity) response design.

Estimated regression coefficients for the response surface design			
Term	Coefficient	Standard error of the coefficient	*p*-value
Constant	70.981	4.665	0.000
w(ME), %	1.703	4.586	0.721
w(GPE), %	12.128	4.586	0.033
w(ME) * *w*(ME)	1.408	6.760	0.841
w(GPE) * *w*(GPE)	14.053	6.760	0.076
w(ME) * *w*(GPE)	7.727	5.617	0.211

Analysis of variance (ANOVA) for response surface model (coded units)						
Source of variation	Degrees of freedom	Sequential sum of squares	Adjusted sum of squares	Adjusted mean of squares	*F*-value	*p*-value
Regression	5	1831.93	1831.93	366.386	2.90	0.099
Linear	2	899.99	899.99	449.993	3.57	0.086
Square	2	693.09	693.09	346.544	2.75	0.132
Interaction	1	238.86	238.86	238.857	1.89	0.211
Residual Error	7	883.43	883.43	126.205		
Lack-of-Fit	3	808.91	808.91	269.638	14.47	0.013
Pure Error	4	74.52	74.52	18.630		
Total	12	2715.36				

Table S6. Estimated regression coefficients and analysis of variance for DPPH* (DPPH* radical scavenging activity) response design.

Estimated regression coefficients for the response surface design

Term	Coefficient	Standard error of the coefficient	*p*-value
Constant	55.9224	2.930	0.000
w(ME), %	-5.5683	2.880	0.094
w(GPE), %	12.9533	2.880	0.003
w(ME) * *w*(ME)	3.2516	4.245	0.469
w(GPE) * *w*(GPE)	-5.7334	4.245	0.219
w(ME) * *w*(GPE)	-0.9500	3.528	0.795

Analysis of variance (ANOVA) for response surface model (coded units)

Source of variation	Degrees of freedom	Sequential sum of squares	Adjusted sum of squares	Adjusted mean of squares	*F*-value	*p*-value
Regression	5	1290.85	1290.85	258.17	5.19	0.026
Linear	2	1192.77	1192.77	596.39	11.98	0.005
Square	2	94.47	94.47	47.24	0.95	0.432
Interaction	1	3.61	3.61	3.61	0.07	0.795
Residual Error	7	348.43	348.43	49.78		
Lack-of-Fit	3	147.15	147.15	49.05	0.97	0.488
Pure Error	4	201.28	201.28	50.32		
Total	12	1639.28				

Table S7. Estimated regression coefficients and analysis of variance for ferric-reducing power (FRP) response design.

Estimated regression coefficients for the response surface design

Term	Coefficient	Standard error of the coefficient	*p*-value
Constant	51.6169	3.340	0.000
w(ME), %	2.6083	3.284	0.453
w(GPE), %	9.2333	3.284	0.026
w(ME) * *w*(ME)	0.6309	4.840	0.900
w(GPE) * *w*(GPE)	1.2359	4.840	0.806
w(ME) * *w*(GPE)	-3.4250	4.022	0.423

Analysis of variance (ANOVA) for response surface model (coded units)

Source of variation	Degrees of freedom	Sequential sum of squares	Adjusted sum of squares	Adjusted mean of squares	*F*-value	*p*-value
Regression	5	607.41	607.409	121.482	1.88	0.217
Linear	2	552.35	552.347	276.174	4.27	0.061
Square	2	8.14	8.139	4.070	0.06	0.940
Interaction	1	46.92	46.923	46.923	0.73	0.423
Residual Error	7	452.88	452.882	64.697		
Lack-of-Fit	3	447.79	447.785	149.262	117.14	0.000
Pure Error	4	5.10	5.097	1.274		
Total	12	1060.29				

Table S8. Estimated regression coefficients and analysis of variance for ferrous chelating capacity (CHE) response design.

Estimated regression coefficients for the response surface design

Term	Coefficient	Standard error of the coefficient	*p*-value
Constant	196.817	11.64	0.000
w(ME), %	0.828	11.44	0.944
w(GPE), %	-2.182	11.44	0.854
w(ME) * *w*(ME)	8.777	16.87	0.619
w(GPE) * *w*(GPE)	5.397	16.87	0.758
w(ME) * *w*(GPE)	-1.720	14.02	0.906

Analysis of variance (ANOVA) for response surface model (coded units)

Source of variation	Degrees of freedom	Sequential sum of squares	Adjusted sum of squares	Adjusted mean of squares	*F*-value	*p*-value
Regression	5	504.11	504.11	100.82	0.13	0.981
Linear	2	32.67	32.67	16.34	0.02	0.979
Square	2	459.60	459.60	229.80	0.29	0.755
Interaction	1	11.83	11.83	11.83	0.02	0.906
Residual Error	7	5500.42	5500.42	785.77		
Lack-of-Fit	3	393.90	393.90	131.30	0.10	0.954
Pure Error	4	5106.52	5106.52	1276.63		
Total	12	6004.53				

Table S9. Second-order polynomial equations and regression coefficient of the response variables*.

Response	Regression equation	R^2 (%)
TPC (mg GAE/100 mL)	$y = 57.18 + 19.48 \cdot w(\text{GPE}) + \varepsilon$	69.93
ABTS*+ (mg Trolox/100 mL)	$y = 70.98 + 12.12 \cdot w(\text{GPE}) + \varepsilon$	67.47
DPPH* (mg Trolox/100 mL)	$y = 55.92 + 12.95 \cdot w(\text{GPE}) + \varepsilon$	78.74

Total phenolic content – TPC; ABTS+ scavenging activity - ABTS*+; DPPH* scavenging activity- DPPH*

Table S10. Data set for overall optimization*

Response	Goal	Lower	Target	Upper
TPC	Maximum	73	99	99
ABTS*+	Maximum	78	109	109
DPPH*	Minimum	65	65	74

Total phenolic content – TPC; ABTS+ scavenging activity - ABTS*+; DPPH* scavenging activity- DPPH*

Table S11. The polypeptide composition of the optimized milk/ME/GPE and control TM powders, analyzed by SDS-PAGE in reducing conditions (SDS-R-PAGE).

Polypeptide	**TM** (% total proteins)	**Milk/ME/GPE** (% total proteins)
Lf	3.10	2.52
BSA	5.30	6.58
Ighc	3.73	4.13
αS2-CN	13.98	12.09
αS1-CN	6.25	4.94
β-CN	16.70	11.76
κ-CN	9.34	6.45
*	/	3.75
*	/	3.07
βLG	11.53	9.47
paraκ-CN	/	7.42
αLA	10.75	9.14

Abbreviations: Lactoferrin (Lf); bovine serum albumin (BSA); Imunoglobulin heavy chain (Ighc); αs2-casein (αs2-CN); αs1-casein (αs1-CN); β-casein (β-CN); κ-casein (κ-CN); β-lactoglobulins (β-LG); paraκ-casein (paraκ-CN); α-lactalbumins (α-LA). * low molecular weight polypeptides.

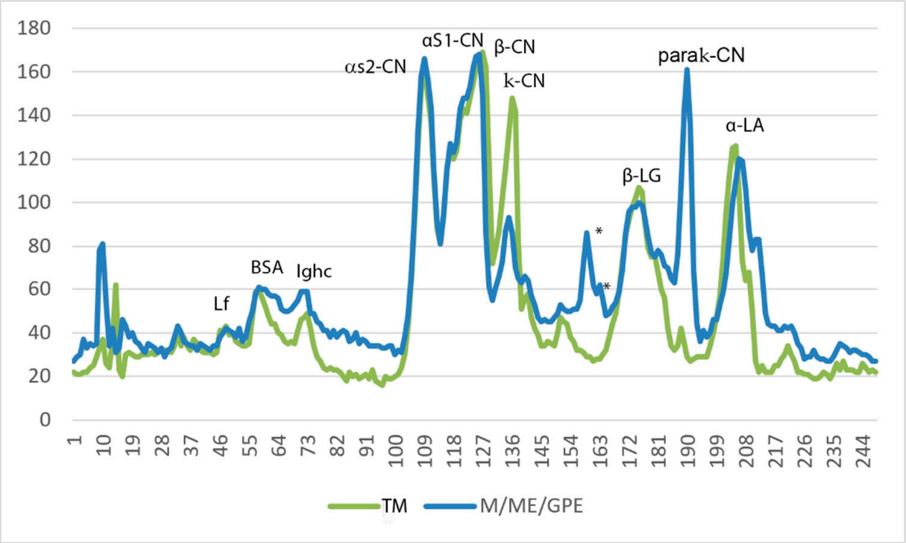

Figure S1. Electrophoregrams of polypeptide profile of the optimized milk/ME/GPE and control TM powders, analyzed by SDS-PAGE in reducing conditions (SDS-R-PAGE).
